# Dynamic prediction of slope displacement using Vmd decomposition with collaborative lssvm-lstm optimization

**DOI:** 10.1038/s41598-025-19081-9

**Published:** 2025-10-08

**Authors:** Miren Rong, Chao Feng, Hailong Wang, Lanxin Luo, Ying Yuan, Dongyang Geng, Jiayin Li

**Affiliations:** 1 School of Urban Geology and Engineering, Hebei GEO University, Shijiazhuang, 052161 China; 2 Hebei Technology Innovation Center for Intelligent Development and Control of Underground Built Environment, Shijiazhuang, 052161 China; 3 Shijiazhuang Tiedao University, Shijiazhuang, 050043 China

**Keywords:** VMLL, Slope displacement prediction, Landslide early warning, Variational mode decomposition, Marine predators algorithm, High-fill embankment slope, Engineering, Environmental sciences, Natural hazards, Solid Earth sciences

## Abstract

With the in-depth implementation of China’s “National Strategy for Building a Strong Transportation Network,” the scale of expressway construction has continued to expand. As a result, the number of high-fill and deep-cut subgrade projects under complex geological conditions has increased significantly, leading to a surge in landslide-related issues. Consequently, accurate prediction of slope displacement is of critical importance for early warning and prevention of landslide disasters. This study proposes a hybrid prediction model, VMD-MPA-LSSVM-LSTM (VMLL), which integrates Variational Mode Decomposition (VMD), Marine Predators Algorithm (MPA), Least Squares Support Vector Machine (LSSVM), and Long Short-Term Memory (LSTM) networks. Using monitoring data from the high-fill embankment slope at Hongtuyao as the research subject, the VMLL model is employed to predict slope displacement based on small-sample data. The objective is to provide a more accurate method for early warning of slope displacement. Firstly, the original monitoring data are decomposed into trend displacement components and fluctuation displacement components using Variational Mode Decomposition (VMD). Subsequently, the trend component and the fluctuation component are predicted using Least Squares Support Vector Machine (LSSVM) and Long Short-Term Memory (LSTM) networks, respectively. Finally, the Marine Predators Algorithm (MPA) is employed to optimize the hyperparameters of the predictive models. Based on this framework, a VMLL-based slope displacement prediction model is constructed. To verify the superiority of the VMLL model, a comparative analysis was conducted against LSSVM, LSTM, and the VMD-LSSVM-LSTM models. The results demonstrate that the VMLL model achieves the highest prediction accuracy, with a Mean Absolute Percentage Error (MAPE) of 0.4022%, a Mean Absolute Error (MAE) of 0.016 mm, a Root Mean Square Error (RMSE) of 0.0206 mm, a coefficient of determination (R²) of 94.08%, and a Variance Accounted For (VAF) of 96.5%. Compared with the other three models, the VMLL model reduces the MAPE, MAE, and RMSE by 30.41–67.62%, 30.38–67.40%, and 27.32–71.00%, respectively. Meanwhile, it improves the R² and VAF by 5.95–217.51% and 0.38–11.48%, respectively. These improvements clearly demonstrate that the VMLL model outperforms the other models, indicating its significant advantage over single prediction models. Furthermore, three additional datasets were used to evaluate the model’s performance. The average values of the performance evaluation metrics for these datasets were 0.6207% (MAPE), 0.0238 mm(MAE), 0.0273 mm(RMSE), 90.33%(R²), and 96.81%(VAF), respectively. These results demonstrate the high accuracy and strong robustness of the proposed model in predicting both horizontal displacement and vertical settlement of slopes, providing a reliable methodological framework for slope stability assessment and landslide disaster early warning.

## Introduction

Slope stability has long been a critical research topic in the field of geotechnical engineering^1^especially in major infrastructure projects such as highways, where slope failures leading to landslides pose serious threats to the safety of lives and property^2–4^. In recent years, slope disasters have occurred frequently, with landslides on the Hubei Expressway, collapses on the Meida Expressway, and landslides in Cangnan causing significant casualties. These incidents not only resulted in severe loss of life but also highlighted the limitations of traditional monitoring methods^5^. Against this backdrop, effective slope risk early warning is of great significance^6,7^and the development of novel slope disaster prediction methods has become an inevitable choice to enhance engineering safety and disaster prevention capabilities^8^.

Changes in slope displacement effectively reflect landslide instability. As a core indicator for assessing landslide risk^9^its monitoring and prediction methods have undergone systematic advancements. Traditional prediction methods primarily rely on physically driven models^10^. Bing-Liu^11^ and colleagues employed numerical simulation using the Discrete Element Method (DEM) to thoroughly investigate the deformation mechanism of fractured rock slopes during the reservoir filling process of the Xiluodu Dam. Their study confirmed that the coupling effect between changes in pore water pressure and the weakening of rock mass parameters is the key factor leading to shear slip; Park-Hyeok-Jin^12^ conducted a systematic review of the application progress of hydrological-mechanical coupled models such as SHALSTAB^13^SINMAP^14^and TRIGRS^15^. These models significantly improve the accuracy of landslide susceptibility assessments by integrating hydrological processes with slope stability analysis. Meanwhile, significant breakthroughs have been made in statistical inference methods. For example, the Bayesian updating method developed by Dian-Qing-Li’s team^16^ effectively reduces the uncertainty in rock mass parameters and displacement prediction by integrating field monitoring data, resulting in a marked improvement in the agreement between predicted and observed data. Although these methods have achieved significant success in mechanism interpretation and stability assessment, their ability to model complex nonlinear displacement time series remains limited, and they struggle to accommodate the high-noise characteristics of field monitoring data. This opens new research avenues for data-driven machine learning approaches.

To overcome the limitations of traditional methods, machine learning techniques have, in recent years, demonstrated remarkable advantages in slope displacement prediction due to their strong nonlinear modeling capabilities and adaptability to diverse data^17^. At present, commonly used artificial intelligence algorithms for landslide prediction include neural networks^18–21^decision trees^22,23^support vector machines^24–26^and grey models^27^. In terms of algorithmic innovation, researchers have made significant progress by integrating intelligent optimization with machine learning. Xiao^28^ integrated the particle swarm optimization (PSO) algorithm with a backpropagation (BP) neural network, and the resulting PSO-BP model significantly improved the prediction accuracy of infiltration depth in loess slopes; Wei et al.^29^ innovatively combined the whale optimization algorithm with the Harris hawks optimization algorithm to enhance the support vector regression model, achieving a performance breakthrough in slope safety factor prediction. Mohammad^30^ proposed the GBAEF-SVR hybrid model, which further improved prediction accuracy through a global optimization strategy. These studies demonstrate that the organic integration of intelligent optimization algorithms with traditional machine learning models can effectively enhance the accuracy and reliability of slope prediction.

While machine learning methods continue to advance slope prediction technologies, data quality has increasingly become a critical factor limiting further improvements in model performance. During slope monitoring, displacement data collected are often affected by varying degrees of noise due to environmental disturbances, equipment errors, and other factors. To address this issue, researchers have conducted systematic explorations from the perspectives of data preprocessing and model optimization. In terms of data processing, Shen^31^ demonstrated through wavelet transform combined with finite element analysis that denoising can significantly improve the reliability of seismic response analysis for soil slopes; Jiang^32^ performed feature selection and dimensionality reduction on multi-source monitoring data using Pearson correlation analysis and grey relational analysis, effectively optimizing the quality of input data and significantly improving the accuracy of rock slope displacement prediction; Shao and Liu^33^ proposed a slope deformation prediction model based on deep learning and Gaussian filtering, which improves the accuracy and reliability of slope deformation predictions through noise and probabilistic analysis. These studies provide an effective technical approach for handling random disturbances in monitoring data. In terms of model optimization, Guoqing Ma employed a wavelet decomposition (WD) and improved particle swarm optimization (IPSO) enhanced gated recurrent unit (GRU) to accurately predict the surface displacement of bedrock-covered slopes during tunnel construction; Yao^34^ applied a sparrow search algorithm with an irrational escape strategy (IESSA) to optimize the least squares support vector machine (LSSVM), which not only improved prediction accuracy but also enhanced the model’s robustness to noisy data. These studies indicate that the integration of signal processing techniques with intelligent algorithms can more effectively handle noisy monitoring data, providing more reliable technical support for slope stability analysis.

In summary, although artificial intelligence algorithms have made significant progress in the field of slope prediction, several key issues remain insufficiently addressed. Firstly, single models exhibit limited generalization ability when dealing with complex nonlinear and non-stationary displacement sequences, making it difficult to adequately capture both the long-term trends and sudden local variations of slope deformation. Secondly, most machine learning approaches are sensitive to the high noise inherent in monitoring data and lack effective mechanisms for separating fluctuations from trends, which renders their predictions easily disturbed by abnormal variations. Moreover, existing hybrid models tend to focus on optimization at a single level (such as parameter tuning or data preprocessing), without systematically integrating the advantages of signal decomposition, multi-model collaboration, and optimization algorithms, and thus have not yet formed a robust framework for slope displacement prediction.

To address the above research gaps, a hybrid prediction model termed VMLL(VMD-MPA-LSSVM-LSTM) is proposed. First, the variational mode decomposition(VMD) is employed to adaptively decompose the original displacement series, effectively separating the trend and fluctuation components while suppressing noise and preserving essential deformation features. Then, a collaborative prediction framework combining LSSVM and LSTM is constructed, where LSSVM is used to capture the trend component and LSTM is applied to model the fluctuation component. Finally, the marine predators algorithm(MPA) is utilized to synchronously optimize the critical hyperparameters of both LSSVM and LSTM, thereby yielding the final VMLL prediction model. To comprehensively evaluate the model performance, MAPE, MAE, RMSE, R², and VAF are introduced as evaluation metrics. Finally, a real slope engineering case is employed to verify the accuracy and predictive capability of the proposed model, thereby achieving precise prediction of slope displacement.

## Methodology

### Variational mode decomposition

Variational Mode Decomposition (VMD) is an adaptive signal decomposition method^35^. Its core idea is to decompose a signal $\:x\left(t\right)$ into $\:K$ mode functions $\:{u}_{k}\left(t\right)(k=\text{1,2},\dots\:,K)$ by solving a variational problem, where each mode is compactly distributed around its central frequency $\:{\omega\:}_{k}$ in the frequency domain. Compared with the traditional Empirical Mode Decomposition (EMD), VMD avoids the problem of mode mixing and is supported by a more solid mathematical foundation.

The variational problem formulation and constraints of VMD are as follows:

1 $$\mathop {\hbox{min} }\limits_{{\left\{ {{u_k}} \right\},\left\{ {{\omega _k}} \right\}}} \left\{ {\sum\limits_{{k=1}}^{K} {{\partial _t}} \left[ {\left( {\delta \left( t \right)+\frac{j}{{\pi t}}} \right)*{u_k}\left( t \right)} \right]{e^{ - j{\omega _k}t_{2}^{2}}}} \right\}$$

2 $$\mathop \sum \limits_{{k = 1}}^{K} u_{k} \left( t \right) = x\left( t \right)$$

In the formula, $\:{\partial\:}_{t}$ denotes the time derivative, $\:\delta\:\left(t\right)$ is the Dirac delta function, $\:*$ represents the convolution operation, and $\:j$ is the imaginary unit. The optimization is then solved using the Alternating Direction Method of Multipliers (ADMM), with the main iterative steps as follows:

①Update of modal functions

3 $$\hat {u}_{k}^{{n+1}}\left( \omega \right)=\frac{{\hat {x}\left( \omega \right) - \sum\nolimits_{{i \ne k}} {{{\hat {u}}_i}} \left( \omega \right)+\frac{{\hat {\lambda }\left( \omega \right)}}{2}}}{{1+2\alpha {{(\omega - {\omega _k})}^2}}}$$

②Update of central frequencies

4 $$\omega _{k}^{{n + 1}} = \frac{{\mathop \smallint \nolimits_{0}^{\infty } \omega |\hat{u}_{k} \left( \omega \right)|^{2} d\omega }}{{\mathop \smallint \nolimits_{0}^{\infty } |\hat{u}_{k} \left( \omega \right)|^{2} d\omega }}$$

③Update of Lagrange multipliers

5 $${\hat {\lambda }^{n+1}}\left( \omega \right)={\hat {\lambda }^n}\left( \omega \right)+\tau \left( {\hat {x}\left( \omega \right) - \mathop \sum \limits_{{k=1}}^{K} \hat {u}_{k}^{{n+1}}\left( \omega \right)} \right)$$

Here, $$\:{\widehat{u}}_{k}\left(\omega\:\right)$$$$\:\widehat{x}\left(\omega\:\right)$$, and $$\:\widehat{\lambda\:}\left(\omega\:\right)$$ are the Fourier transforms of $$\:{u}_{k}\left(t\right)$$$$\:x\left(t\right)$$, and *λ*(*t*), respectively, and $$\:\tau\:$$ is the update step size.

Let $\:ϵ$ be the convergence threshold, and iterate until the convergence condition is satisfied:

6 $$\mathop \sum \limits_{{k=1}}^{K} \frac{{\left\| {\hat {u}_{k}^{{n+1}} - \hat {u}_{k}^{n}} \right\|_{2}^{2}}}{{\left\| {\hat {u}_{k}^{n}} \right\|_{2}^{2}}}< \in$$

### Least squares support vector machine

The Least Squares Support Vector Machine (LSSVM)^36^ is a variant of the Support Vector Machine (SVM)^37^. By transforming inequality constraints into equality constraints, it converts the original quadratic programming problem into a linear system, significantly improving computational efficiency and making it more suitable for handling small-sample data and nonlinear problems^38^.

For displacement time series data of length $\:T$, the training sample set $\:{\left\{\left({X}_{t},{y}_{t}\right)\right\}}_{t=k}^{T-1}$ is constructed using the sliding window method, where the input features are:

7 $$\mathop {\min }\limits_{{w,b,e}} \frac{1}{2}\left\| w \right\|^{2} + \frac{\gamma }{2}\mathop \sum \limits_{{t = k}}^{{T - 1}} e_{t}^{2}$$

The constraint is:

8 $${y_t}={w^ \top }\phi \left( {{X_t}} \right)+b+{e_t},t=k, \ldots ,T - 1$$

Here, $\:w$ is the weight vector; $\:\varphi\:\left({X}_{t}\right)$ is the nonlinear mapping function; $\:\gamma\:$ is the regularization parameter (balancing model complexity and training error); and $\:{e}_{t}$ is the training error for the t sample.

By solving the linear system in the dual space, the analytical solution is obtained:

9 $$\left[ {\begin{array}{*{20}{c}} 0&{{1^T}} \\ 1&{K+{\gamma ^{ - 1}}I} \end{array}} \right]\left[ {\begin{array}{*{20}{c}} b \\ \alpha \end{array}} \right]=\left[ {\begin{array}{*{20}{c}} 0 \\ y \end{array}} \right]$$

The elements of the kernel matrix $\:K$ satisfy $\:{K}_{i,j}={\varphi\:\left({X}_{i}\right)}^{T}\varphi\:\left({X}_{j}\right)=\mathcal{K}\left({X}_{i},{X}_{j}\right)$, and the radial basis function (RBF) is used as the kernel: $\:\mathcal{K}({X}_{i},{X}_{j})=\text{e}\text{x}\text{p}(-\parallel\:{X}_{i}-{X}_{j}{\parallel\:}^{2}/2{\sigma\:}^{2})$. The single-step prediction model is finally realized:

10 $$f\left( X \right) = \sum\limits_{{t = k}}^{{T - 1}} {\alpha _{t} } {\text{ }}{\mathcal{K}}\left( {X,X_{t} } \right) + b$$

### Long short-term memory

Long Short-Term Memory network (LSTM)^39^ is a special type of Recurrent Neural Network (RNN) that effectively addresses the vanishing and exploding gradient problems of traditional RNNs through a gating mechanism. Its core structure consists of memory cells and three gates (input gate, forget gate, output gate), which dynamically control the information flow, making it particularly suitable for time series prediction tasks such as slope displacement forecasting^40,41^.

Gating mechanism:

11 $$\left\{ {\begin{array}{*{20}{c}} {{i_\tau }=\sigma \left( {{W_i} \cdot \left[ {{h_{\tau - 1}},X_{t}^{{\left( \tau \right)}}} \right]+{b_i}} \right)} \\ {{f_\tau }=\sigma \left( {{W_f} \cdot \left[ {{h_{\tau - 1}},X_{t}^{{\left( \tau \right)}}} \right]+{b_f}} \right)} \\ {{0_\tau }=\sigma \left( {{W_o} \cdot \left[ {{h_{\tau - 1}},X_{t}^{{\left( \tau \right)}}} \right]+{b_o}} \right)} \end{array}} \right.$$

State update:

12 $$\left\{ {\begin{array}{*{20}{c}} {{{\tilde {C}}_\tau }=\tanh \left( {{W_C} \cdot \left[ {{h_{\tau - 1}},X_{t}^{{\left( \tau \right)}}} \right]+{b_C}} \right)} \\ {{C_\tau }={f_\tau } \cdot {C_{\tau - 1}}+{i_\tau } \cdot {{\tilde {C}}_\tau }} \\ {{h_\tau }={o_\tau } \cdot \tanh \left( {{C_\tau }} \right)} \end{array}} \right.$$

Here, $\:\sigma\:$ denotes the Sigmoid function, and the output layer maps the state through a fully connected layer:

13 $${\hat {y}_t}={W_y}{h_k}+{b_y}$$

During training, the Adam optimizer is used to minimize the mean squared error loss:

14 $$\mathcal{L}=\frac{1}{{{N_{train}}}}\mathop \sum \limits_{{\left( {{X_t},{y_t}} \right) \in Train}} {\left( {{y_t} - {{\hat {y}}_t}} \right)^2}+\lambda W_{2}^{2}$$

### Marine predators algorithm

MPA is a metaheuristic optimization algorithm inspired by predator–prey interactions in nature, proposed by Faramarzi^42^ in 2020. The main idea mimics the hunting behavior of top predators^43,44^where the top predators form an elite matrix (each top predator represents a solution to the problem). By combining Lévy Flight^45^ and Brownian Motion^46^the algorithm achieves a balance between global exploration and local exploitation.

Algorithm Initialization, In a D-dimensional search space, randomly generate N predators (candidate solutions) to form the position matrix X:

15 $${\mathbf{X}}=\left[ {\begin{array}{*{20}{c}} {{x_{1,1}}}&{{x_{1,2}}}& \cdots &{{x_{1,D}}} \\ {{x_{2,1}}}&{{x_{2,2}}}& \cdots &{{x_{2,D}}} \\ \vdots & \vdots & \ddots & \vdots \\ {{x_{N,1}}}&{{x_{N,2}}}& \cdots &{{x_{N,D}}} \end{array}} \right]$$

In the equation, $\:{x}_{i,j}$ represents the position of the i-th predator in the j-th dimension, with initial values uniformly distributed within the search range [lb_j_,ub_j_].

Predator–Prey Interaction Model:

①High-Speed Flight Phase (Early Iterations).

Predators rapidly search for prey using the Lévy flight strategy, and the position update formula is:

16 $${\text{X}}_{i}^{{t+1}}={\text{X}}_{i}^{t}+\alpha \cdot {{\text{R}}_L} \cdot \left( {{\text{Elite}}_{i}^{t} - {\text{X}}_{i}^{t}} \right)$$

In the formula, $\:{\text{R}}_{L}$ is the Lévy flight random vector, $\:\alpha\:$ is the step size factor, and $\:{\text{E}\text{l}\text{i}\text{t}\text{e}}_{i}^{t}$ is the current best solution.

② Equilibrium Movement Phase (Mid Iterations).

Predators and prey move using a combination of Brownian motion and Lévy flight, and the position update formula is:

17 $${\text{X}}_{i}^{{t+1}}={\text{X}}_{i}^{t}+\beta \cdot {{\text{R}}_B} \cdot \left( {{\text{Elite}}_{i}^{t} - {\text{X}}_{i}^{t}} \right)+\left( {1 - \beta } \right) \cdot {{\text{R}}_L} \cdot \left( {{\text{X}}_{r}^{t} - {\text{X}}_{i}^{t}} \right)$$

In the formula, $\:{\text{R}}_{B}$ is the Brownian motion random vector, $\:\beta\:$ is the equilibrium factor, and $\:{\text{X}}_{r}^{t}$ is the position of a randomly selected prey.

③Low-speed movement phase (late iteration):

Predators perform fine local search using the Brownian motion strategy. The position update formula is:

18 $${\text{X}}_{i}^{{t+1}}={\text{X}}_{i}^{t}+\gamma \cdot {{\text{R}}_B} \cdot \left( {{\text{Elite}}_{i}^{t} - {\text{X}}_{i}^{t}} \right)$$

Here, $\:\gamma\:$ is the step-size factor for local search.

Environmental perturbation and adaptive adjustment: to enhance the algorithm’s ability to escape local optima, an environmental perturbation mechanism is introduced:

19 $${\text{X}}_{i}^{{t+1}}={\text{X}}_{i}^{{t+1}}+\eta \cdot {{\text{R}}_E} \cdot \left( {{\text{ub}} - {\text{lb}}} \right)$$

In the formula, $\:{\text{R}}_{E}$ is a random perturbation factor, and $\:\eta\:$ is the perturbation intensity factor.

The algorithm terminates when the maximum number of iterations is reached or the solution quality satisfies the convergence criterion, and the optimal solution is output.

### Creation of the VMLL model

Since field monitoring data are inevitably affected by external disturbances, the raw data consist of a series of nonlinear and fluctuating displacements. Directly using the raw data for model prediction results in relatively low reliability and fails to accurately reflect the actual field conditions. To address this issue, this study innovatively applies VMD decomposition to achieve a scientific separation of engineering deformations and noise disturbances, rather than merely denoising. Subsequently, LSSVM is used to predict the displacement of the trend component, while LSTM predicts the displacement of the fluctuating component, enabling the model to capture both long-term deformation patterns of the slope and short-term engineering noise characteristics. Finally, the MPA algorithm is employed to optimize and integrate the models, resulting in the VMLL (VMD-MPA-LSSVM-LSTM) slope displacement prediction model. It mainly comprises two aspects, and the two core integration formulas are given as follows:

①Variational mode decomposition and parameter joint optimization formula

20 $$\left\{ {{{\hat {u}}_k},{{\hat {\omega }}_k}} \right\}={\text{arg}}\mathop {min}\limits_{{\left\{ {{u_k}} \right\},\left\{ {{\omega _k}} \right\}}} \left\{ {\mathop \sum \limits_{{k=1}}^{K} {\partial _t}\left[ {\left( {\delta \left( t \right)+\frac{j}{{\pi t}}} \right)*{u_k}\left( t \right)} \right]{e^{ - j{\omega _k}t}}_{2}^{2}+\alpha x\left( t \right) - \mathop \sum \limits_{{k=1}}^{K} {u_k}\left( t \right)_{2}^{2}} \right\}$$

21 $$\left\{ {{\gamma ^*},{\sigma ^*},{W^*},{b^*}} \right\}=MPA\left( {\frac{1}{2}\gamma \mathop \sum \limits_{{i=1}}^{N} e_{i}^{2}+\frac{1}{2}{w^T}w+\mathop \sum \limits_{{t=1}}^{T} \left\| {{y_t} - {W_h}{h_t}} \right\|_{2}^{2}+\lambda \left\| W \right\|_{2}^{2}} \right)$$

Where: The first line is the VMD decomposition process, which decomposes the original signal $\:x\left(t\right)$ into K IMF components $\:{u}_{k}\left(t\right)$ using the Alternating Direction Method of Multipliers; the second line is the MPA optimization process, synchronously optimizing the penalty parameter $\:\gamma\:$ and RBF kernel parameter $\:\sigma\:$ of LSSVM, as well as the weight matrix $\:W$ and bias $\:b$ of LSTM, with the optimization objective including the structural risk minimization of LSSVM and the time series prediction error of LSTM.

② Dual-model prediction integration formula

22 $$\hat {T}\left( t \right)=\mathop \sum \limits_{{i=1}}^{N} {\alpha _i}{\text{exp}}\left( { - \frac{{{{\left\| {{{\bar {u}}_k}\left( t \right) - {X_i}} \right\|}^2}}}{{2{\sigma ^2}}}} \right)+b$$

23 $$\hat {S}\left( t \right)={W_o}\left( {\sigma \left( {{W_f}\left[ {{h_{t - 1}},\sum\limits_{{k=1}}^{K} {{u_k}} \left( t \right)} \right]} \right) \odot \tanh \left( {{C_t}} \right)} \right)+{b_o}$$

24 $$\hat {x}\left( t \right)=\hat {T}\left( t \right)+\hat {S}\left( t \right)$$

Where: the first line is the LSSVM trend component prediction $\:\widehat{T}\left(t\right)$; The second line is the LSTM fluctuation component prediction; The third line is the final integrated prediction formula $\:\widehat{x}\left(t\right)$

**Fig. 1:**
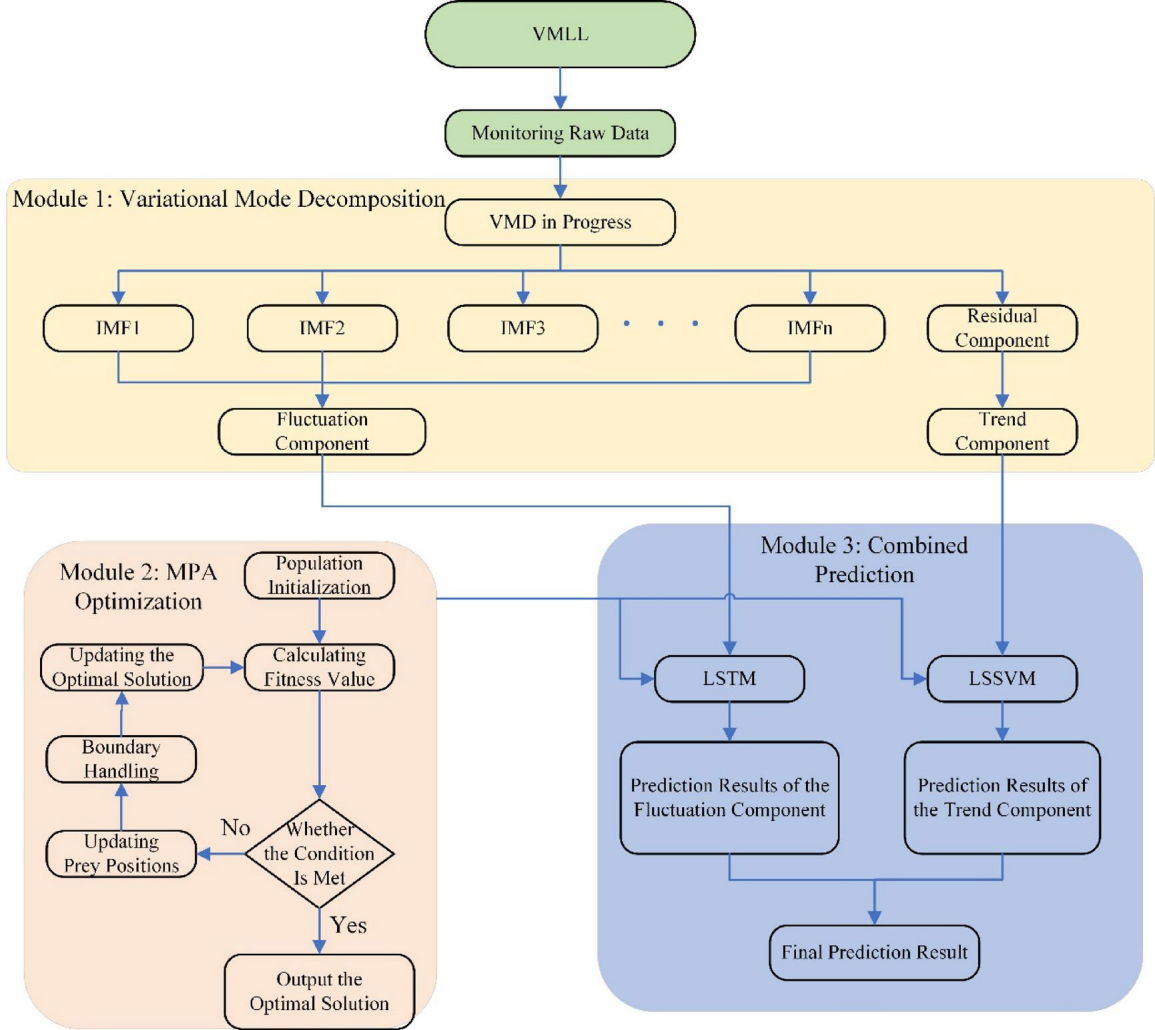
Flowchart of the VMLL Model.

This study implements the VMLL slope prediction model using MATLAB, evaluating its predictive performance from multiple dimensions, and effectively forecasting slope displacement and settlement. Figure 1 shows the flowchart of the VMLL model. The specific implementation steps are as follows:

1. Data preprocessing. The horizontal displacement and settlement data of two slope points were measured using a total station and a Trimble Dini03 digital level. The data were then decomposed using VMD and SPA to obtain trend and fluctuation components, and the optimal decomposition method was determined based on decomposition indicators.
2. Comparison of prediction performance for component combinations. The trend and fluctuation components were predicted using LSSVM and LSTM models in a cross-substitution manner to verify the prediction adaptability of the two models to the trend and fluctuation components.
3. Analysis after model optimization. The MPA was employed to optimize the hyperparameters of both models, and the prediction accuracy was compared with that of the unoptimized models to verify the effectiveness of the optimization algorithm.
  Analysis after model optimization. The MPA was employed to optimize the hyperparameters of both models, and the prediction accuracy was compared with that of the unoptimized models to verify the effectiveness of the optimization algorithm.
4. Small-sample data testing. Predictions were performed using different amounts of data to evaluate the model’s prediction stability under varying training data sizes.
5. Model validation. Since prediction results based on only one dataset may have randomness, multiple datasets from different monitoring points were used to verify the robustness and generalization capability of the VMLL model.

## Engineering background

### Project overview

**Fig. 2:**
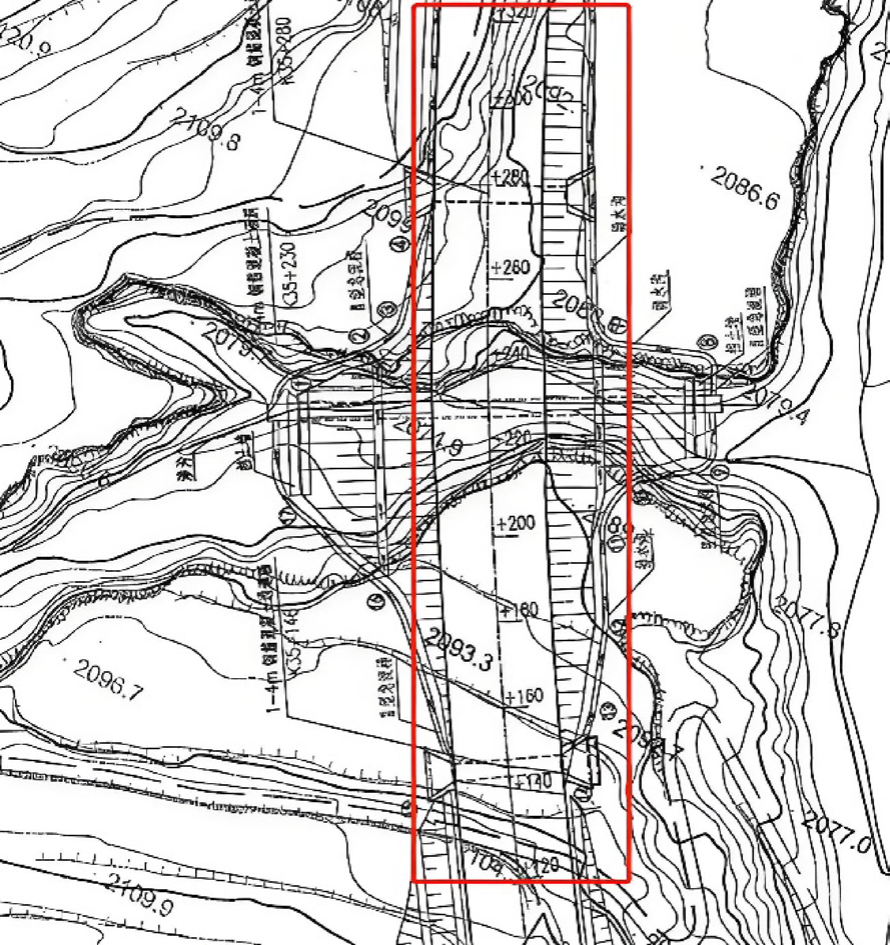
Plan Layout of the High-fill Embankment. (Created by Rong Miren and licensed under CC BY 4.0.)

The Hongtuyao High-fill Embankment, shown in Fig. 2, spans from K35 + 130 to K35 + 318. The stratigraphy consists of aeolian loess, light yellow in color, slightly moist, and moderately dense with high porosity. Vertical joints are developed, exhibiting collapsible soil characteristics. The soil is uniform, primarily silty, with low dry strength, slightly slippery and sticky to the touch. The underlying bedrock is mudstone, brick-red, clay-cemented, poorly lithified, and easily softened when wet. A stepped design is proposed: the first slope is 8 m high with a slope ratio of 1:1.5, protected by a herringbone-shaped framework with grass planting; the second slope is 6 m high with a slope ratio of 1:1.75. The platform width is 2 m. To prevent rainwater erosion on the embankment slopes, prefabricated concrete arch frameworks are used for protection, with grass or shrubbery planted inside the frameworks.

### Layout of monitoring points

**Fig. 3:**
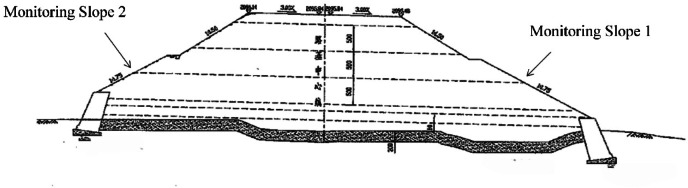
Plan View of Observation Benchmarks. (Created by Rong Miren and licensed under CC BY 4.0.)

Figure 3 presents the plan view of the observation benchmark plane. The maximum fill height of the high-fill embankment slope in this section reaches 16 m, with the slope constructed using a terracing method. Two slope gradients, 1:1.5 and 1:1.75, are planned for this segment. The treated slopes are vegetated with herbaceous plants suitable for local growth to achieve greening. Drainage ditches are installed on each terrace to prevent rainwater erosion. Displacement monitoring points on the slope are arranged along the centerline of the slope width direction, with a spacing of 400 cm along the slope length. These points are symmetrically distributed from the center toward both sides. The monitoring points for horizontal slope displacement and vertical settlement share the same locations.

Settlement monitoring was performed using a Trimble Dini03 digital level combined with a 2-meter Invar leveling rod. A second-order leveling closed loop was established, and height differences between adjacent points along the planned leveling route were measured sequentially. By calculating the height differences relative to a reference point, the elevations of each monitoring point were obtained. The cumulative vertical displacement at each monitoring point was determined by subtracting its initial elevation from the measured elevation. Horizontal displacement was monitored using a total station employing a small-angle measurement method. Reference points were established at a certain distance outside the excavation area, and a baseline was selected. The horizontal displacement monitoring points were arranged as close as possible along this baseline. The theodolite was set up on the reference points to accurately measure the slight angular changes between the baseline and the line of sight from the station to the monitoring points. The horizontal displacement was then calculated from these angular measurements using the corresponding formulas.

The monitoring point data can be classified into normal data and problematic data. Problematic data typically exhibit unstable, random, and noisy behavior. Although such anomalies can often be identified visually, their recognition still requires a degree of subjective judgment. Most of these problematic data arise from measurement defects, human interruptions, or operational errors^47^. To eliminate the influence of these accidental errors, regression processing is carried out on the obtained data to ensure the accuracy of horizontal displacement and numerical settlement displacement, to filter out random fluctuations and reveal the inherent laws of displacement development. The selection function model of commonly used regression functions is as follows:

①Logarithmic function model

This model is suitable for conditions where the displacement rate gradually decreases as time increases. Its expression is:

25 $$u=A{\text{lg}}\left( {1+t} \right)$$

Where $\:u$ denotes the cumulative displacement, $\:t$ represents time, and $\:A$ is the fitting parameter.

②Exponential function model

This model is suitable for processes where displacement changes rapidly and then gradually stabilizes. Its expression is:

26 $$u=A{e^{ - \frac{B}{t}}}$$

Where $\:B$ is the fitting parameter, and $\:e$ is the natural constant.

③Hyperbolic Function Model

This model is widely used to simulate the progressive stabilization of displacement caused by long-term creep of soil, and its expression is:

27 $$u=\frac{t}{{A+Bt}}$$

As shown in the formulas, the three commonly used function models are applied to fit the time series data of the same monitoring point. The goodness of fit is evaluated based on the coefficient of determination (R²), enabling the selection of the trend curve that best represents the displacement variation at that point. This provides a reliable data basis for subsequent data acquisition and analysis.

### Data acquisition

To conduct a reliable displacement time-series prediction, this study selected Slope Monitoring Points 1 and 2. A total of 195 sets of horizontal displacement and vertical settlement data were collected using a total station and Trimble Dini03 digital level. All data were verified on-site by responsible personnel and underwent outlier processing.

**Fig. 4:**
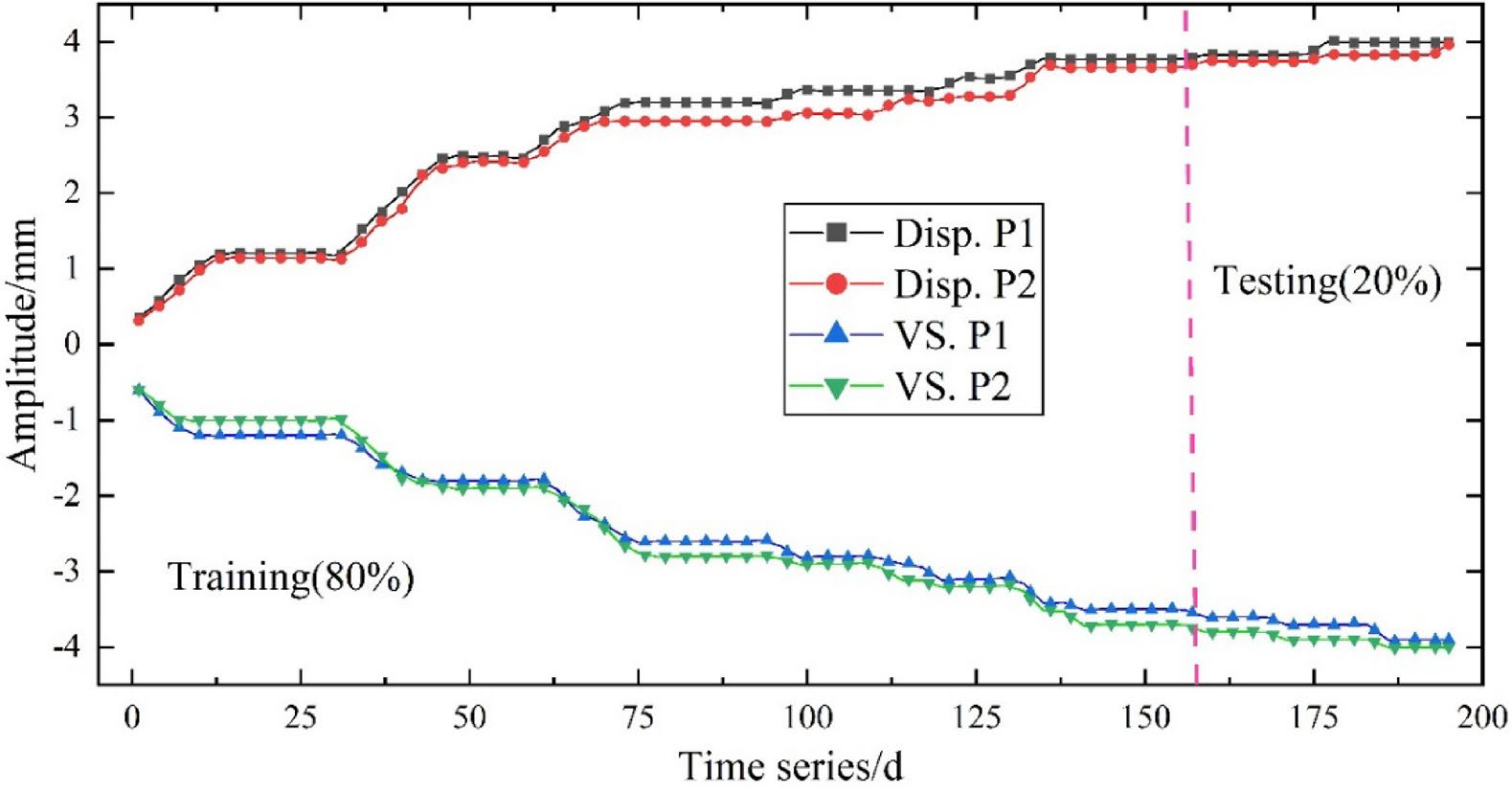
Monitoring data curves.

The final dataset was divided into 80% (156 samples) as the training set and 20% (39 samples) as the testing set, which were used to analyze the characteristics of the learning data and to predict future displacement trends, respectively. As shown in Fig. 4, after the filling operation, the slope was generally in a sub-stable state. The horizontal displacement continued to increase and stabilized after reaching 3.99 mm, while the vertical settlement reached a maximum of 4 mm. This “post-filling accelerated deformation – gradual stabilization after the peak” pattern conforms to the deformation characteristics of sub-stable slopes, and all displacement values were far below the 50 mm warning threshold. The fluctuations in the data not only include short-term abrupt changes caused by construction disturbances but also reflect the continuous influence of environmental variations on the slope under sub-stable conditions, providing a foundation for establishing a VMD decomposition-based predictive model with clear physical significance.

**Fig. 5:**
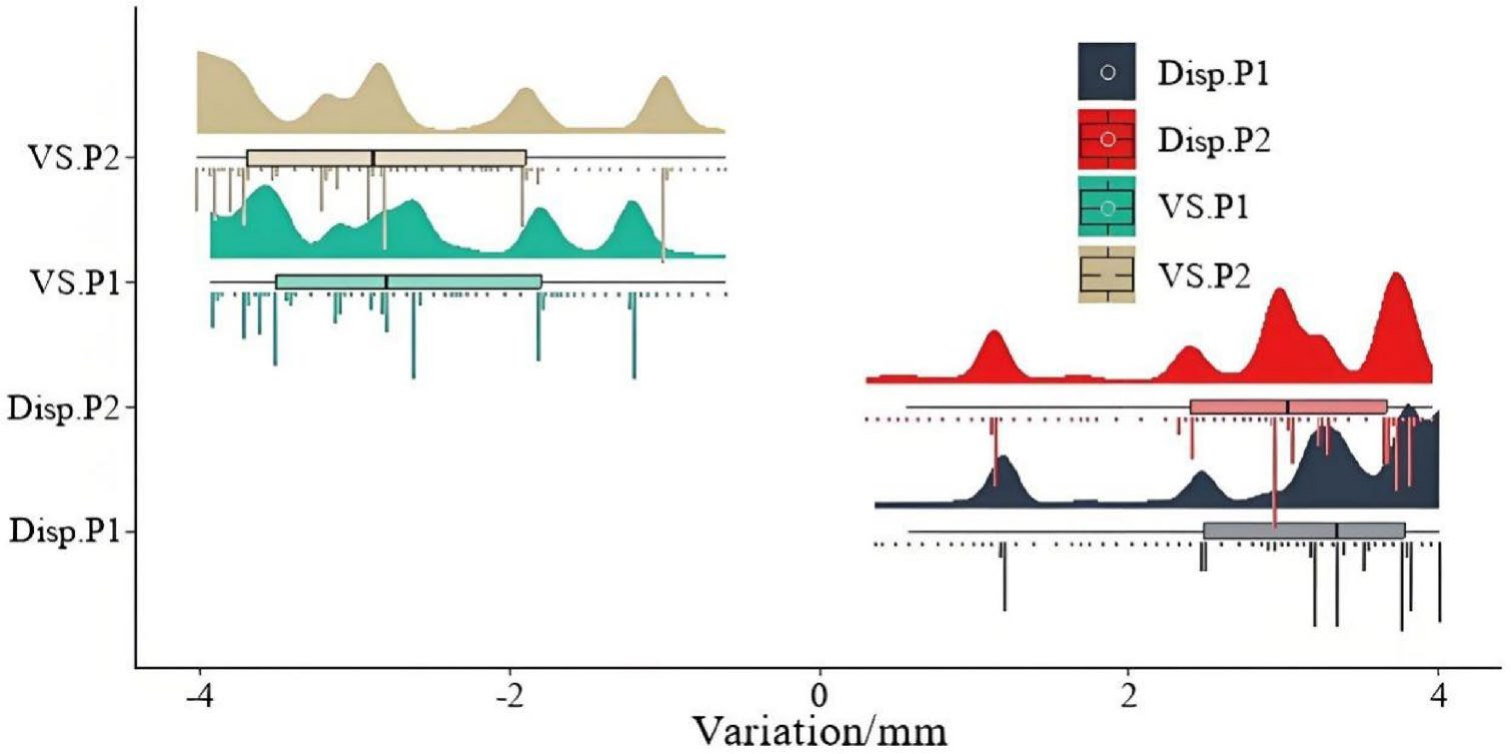
Range and characteristics of data variation.

Figure 5 illustrates the variation ranges of horizontal displacement and vertical settlement at the two monitoring points. From the figure, the following observations can be made: (1) The horizontal displacement data at both monitoring points are relatively concentrated, with a range of 0.36 to 3.99 and a median approximately around 3. Notably, the data distribution for monitoring point 1 is skewed slightly higher, with a greater density of values near the median, better reflecting the typical displacement variations over time; (2) The vertical settlement data for both points exhibit similar distribution patterns, ranging from − 4 to -0.6 with medians around − 2.5. However, the data at monitoring point 1 are more evenly distributed, whereas the data at point 2 are predominantly concentrated near − 2.8, indicating that the settlement variations at point 2 mainly occur during the latter half of the monitoring period; (3) The variation ranges of horizontal displacement and vertical settlement at both points are roughly comparable, indicating a certain degree of spatial correlation between these displacement components.

### Performance evaluation metrics

To comprehensively evaluate the predictive performance of the machine learning models, this study selected five evaluation metrics: Mean Absolute Percentage Error (MAPE), Mean Absolute Error (MAE), Root Mean Square Error (RMSE), Coefficient of Determination (R²), and Variance Accounted For (VAF). These metrics reflect the model’s predictive capability from different perspectives:

MAPE(%) measures the relative error between the predicted and actual values, making it suitable for evaluating prediction accuracy across data of different scales; MAE(mm) reflects the average level of absolute error in predictions and is less sensitive to outliers; RMSE(mm) amplifies the impact of larger errors through squaring, better reflecting prediction stability; R²(%) represents the model’s ability to explain the variance of the target variable, indicating the degree of linear correlation between predicted and actual values; VAF(%) evaluates the model’s capability to capture data variability from the perspective of variance.

This multi-metric evaluation method considers both absolute errors (MAE, RMSE) and relative errors (MAPE), as well as statistical goodness-of-fit (R², VAF), providing a comprehensive and objective assessment of the model’s predictive performance. The ideal values for the three error metrics (MAPE, MAE, RMSE) are 0, while the ideal values for R^2^ and VAF are 100%. The formulas for each metric are as follows:

28 $$MAPE=\frac{1}{N}\mathop \sum \limits_{{i=1}}^{N} \left| {\frac{{y_{i}^{{mea}} - y_{i}^{{pre}}}}{{y_{i}^{{mea}}}}} \right| \times 100\%$$

29 $$MAE=\frac{1}{N}\mathop \sum \limits_{{i=1}}^{N} \left| {y_{i}^{{mea}} - y_{i}^{{pre}}} \right|$$

30 $$RMSE=\sqrt {\frac{1}{N}\mathop \sum \limits_{{i=1}}^{N} {{\left( {y_{i}^{{mea}} - y_{i}^{{pre}}} \right)}^2}}$$

31 $${R^2}=1 - \frac{{\mathop \sum \nolimits_{{i=1}}^{N} {{\left( {y_{i}^{{mea}} - y_{i}^{{pre}}} \right)}^2}}}{{\mathop \sum \nolimits_{{i=1}}^{N} {{\left( {y_{i}^{{mea}} - E\left[ {{y^{mean}}} \right]} \right)}^2}}}$$

32 $$VAF=\left( {1 - \frac{{Var\left( {y_{i}^{{mea}} - y_{i}^{{pre}}} \right)}}{{Var\left( {y_{i}^{{mea}}} \right)}}} \right) \times 100\%$$

33 $${R^2}=1 - \frac{{\mathop \sum \nolimits_{{i=1}}^{N} {{\left( {y_{i}^{{mea}} - y_{i}^{{pre}}} \right)}^2}}}{{\mathop \sum \nolimits_{{i=1}}^{N} {{\left( {y_{i}^{{mea}} - E\left[ {{y^{mean}}} \right]} \right)}^2}}}$$

Where $\:{y}_{i}^{mea}$ is the measured data; $\:{y}_{i}^{pre}$ is the predicted result; $\:E\left[{y}^{mean}\right]$ is the mean of $\:{y}_{i}^{mea}$; *N* is the number of samples; k is the number of independent variables; Var(∙) denotes variance.

## Model prediction analysis

### Data decomposition

To construct a reasonable and effective slope displacement prediction model, preprocessing of the raw data is essential. In practical slope monitoring, measurement instruments are inevitably affected by environmental factors or human operational errors, which introduce erroneous deviations into the raw data and obscure the true variation trends. Therefore, the raw displacement data can be regarded as a combination of trend displacement components and fluctuation displacement components. To effectively separate these components and facilitate targeted analysis, Variational Mode Decomposition (VMD) and the Marine Predators Algorithm (MPA) are employed to decompose the original data into trend and fluctuation components. Subsequently, Least Squares Support Vector Machine (LSSVM) and Long Short-Term Memory (LSTM) networks are applied separately to predict these components, allowing evaluation of which model better captures the characteristics of each component. Here, the horizontal displacement data from monitoring point 1 are used.

①VMD Decomposition.

**Fig. 6:**
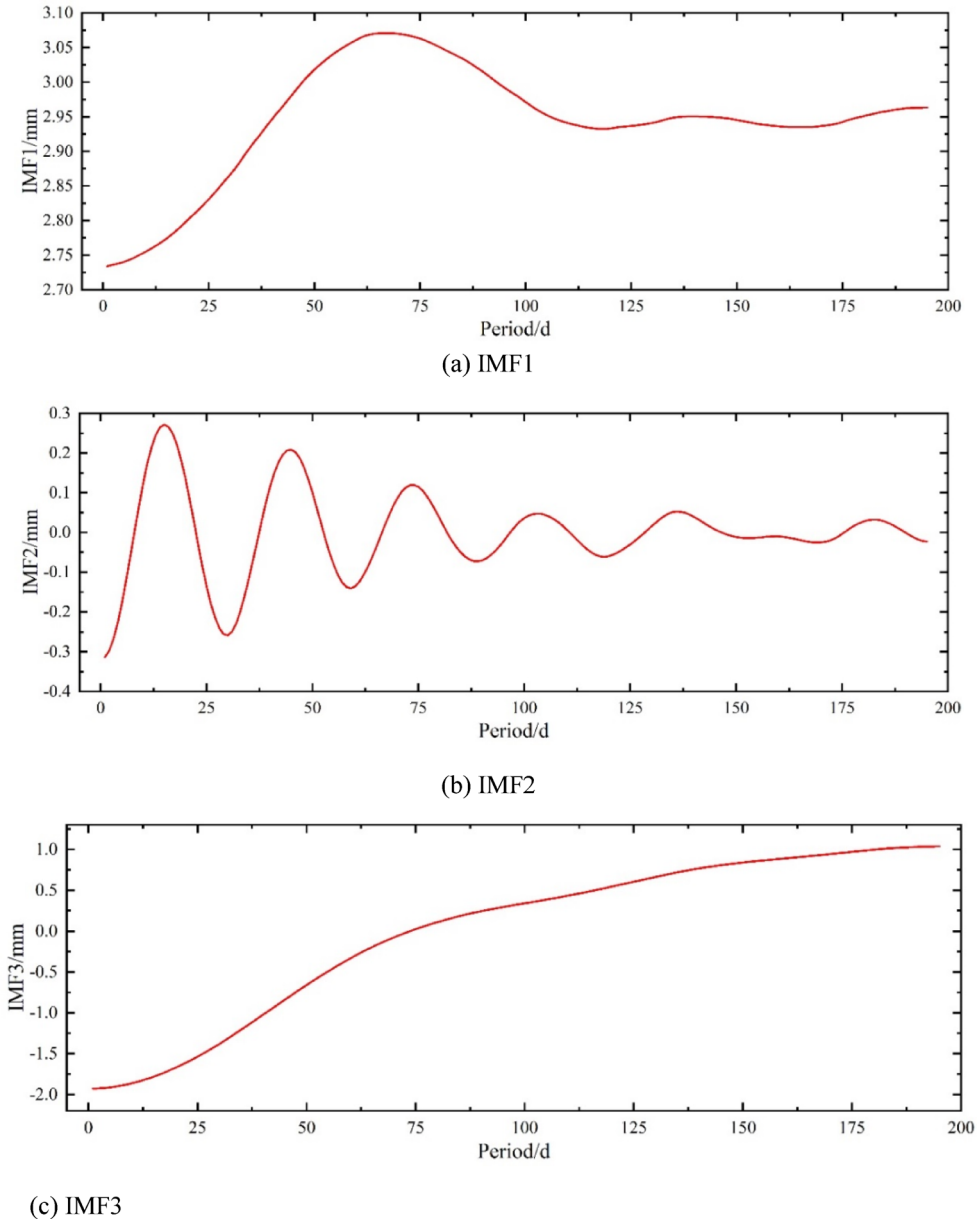

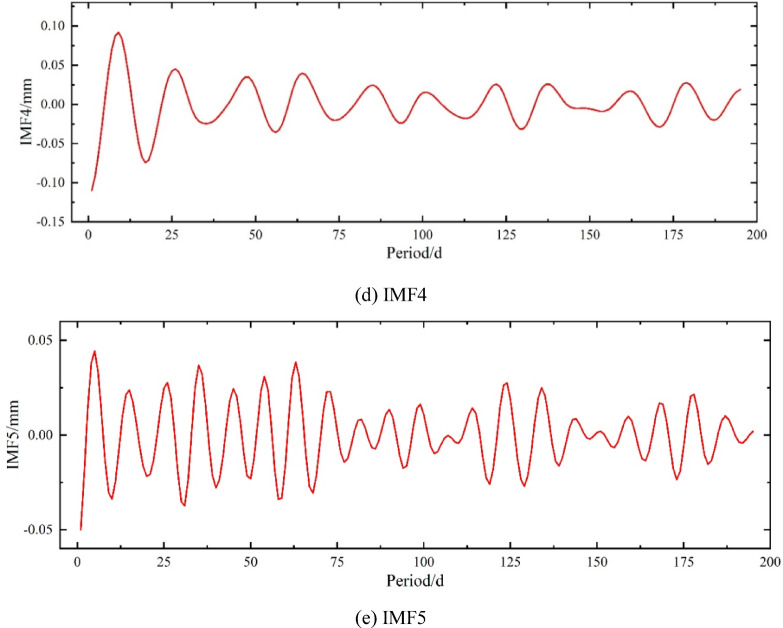
Variation of VMD Components. (**a**) IMF1, (**b**) IMF2, (**c**) IMF3, (**d**) IMF4, (**e**) IMF5.

As shown in Fig. 6, five IMF components (IMF1 ~ IMF5) are obtained through VMD decomposition. It is evident that IMF2, IMF4, and IMF5 exhibit strong fluctuations, oscillating symmetrically around zero at certain high frequencies, demonstrating that the original data are separated into IMF components at different frequencies. IMF1 and IMF3 lack high frequency and fluctuations, so their combination represents the trend component. VMD first separates the trend component, while the fluctuation ranges of the three fluctuation components gradually decrease and their frequencies gradually increase, reflecting the influence of external environmental factors such as temperature, humidity, and human operations. Additionally, the components can be recombined to reconstruct the original data, indicating that the VMD decomposition method is reversible and confirming the completeness of the VMD decomposition.

**Fig. 7:**
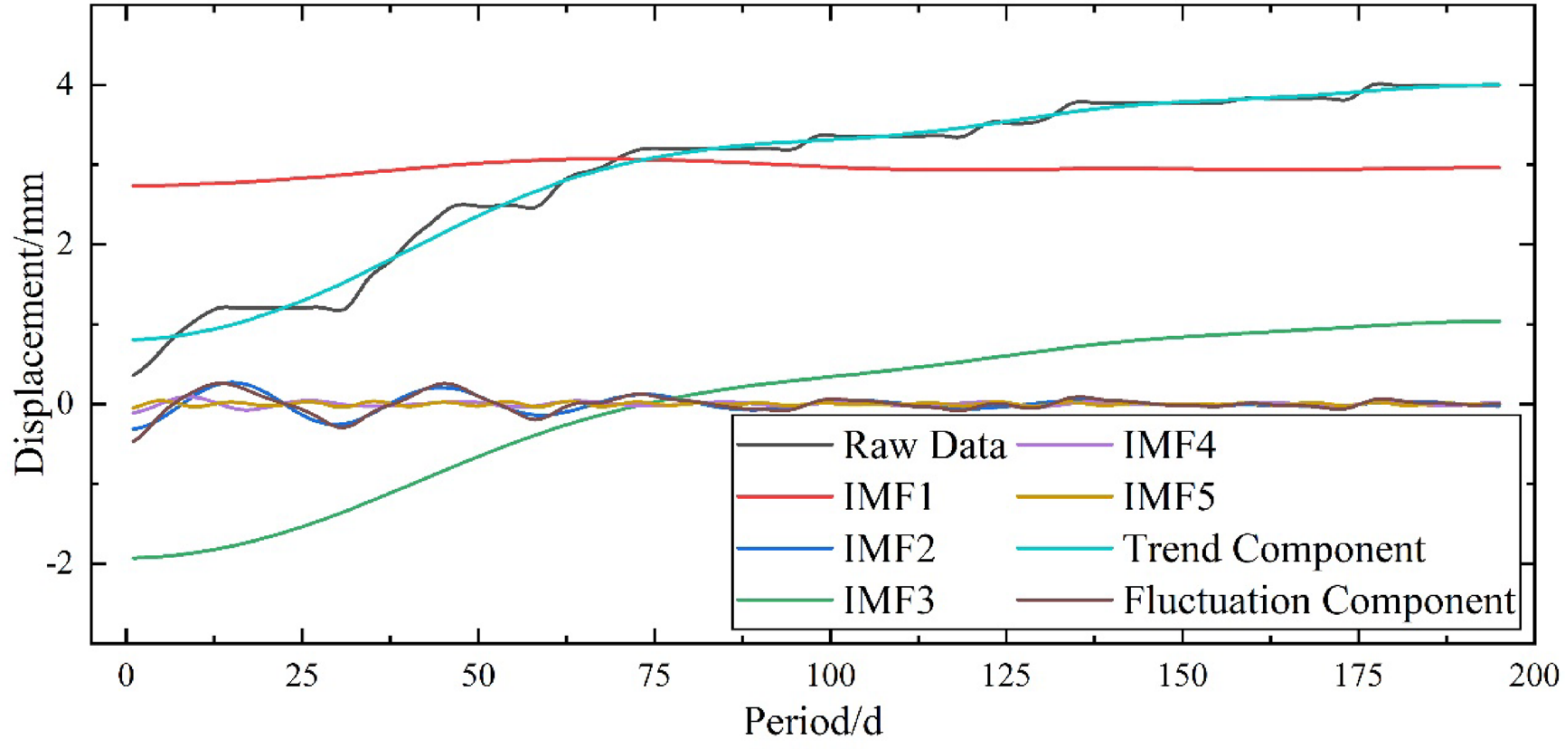
VMD Decomposition and Reconstruction.

As shown in Fig. 7, the VMD decomposition and reconstruction results indicate that IMF(1 + 3) represents the trend component of the original data. The original data show significant fluctuations during the first 100 periods, while fluctuations become milder after the 100th period, and the trend gradually levels off. This behavior is also reflected in the four fluctuation components, where the amplitude of fluctuations becomes gentler. Overall, the trend exhibits a monotonically increasing pattern, which is consistent with the general law of slope settlement changes.

②Smoothing Prior Approach.

**Fig. 8:**
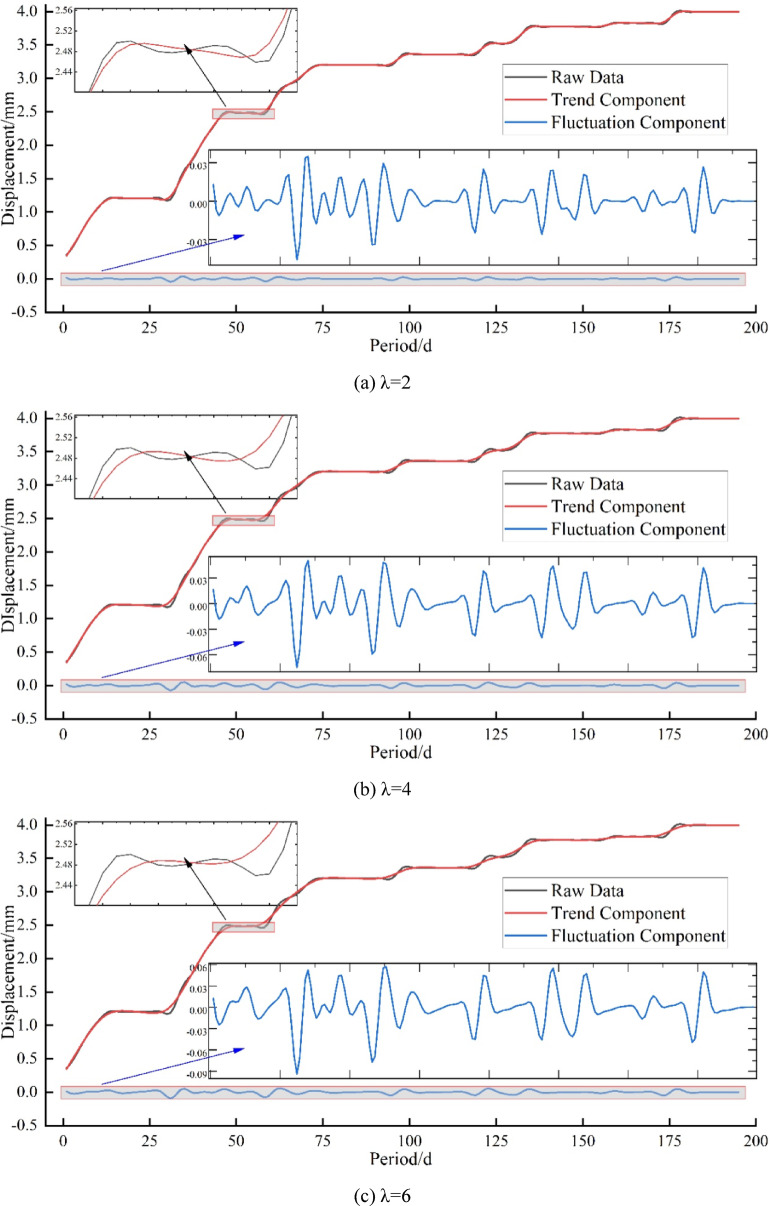

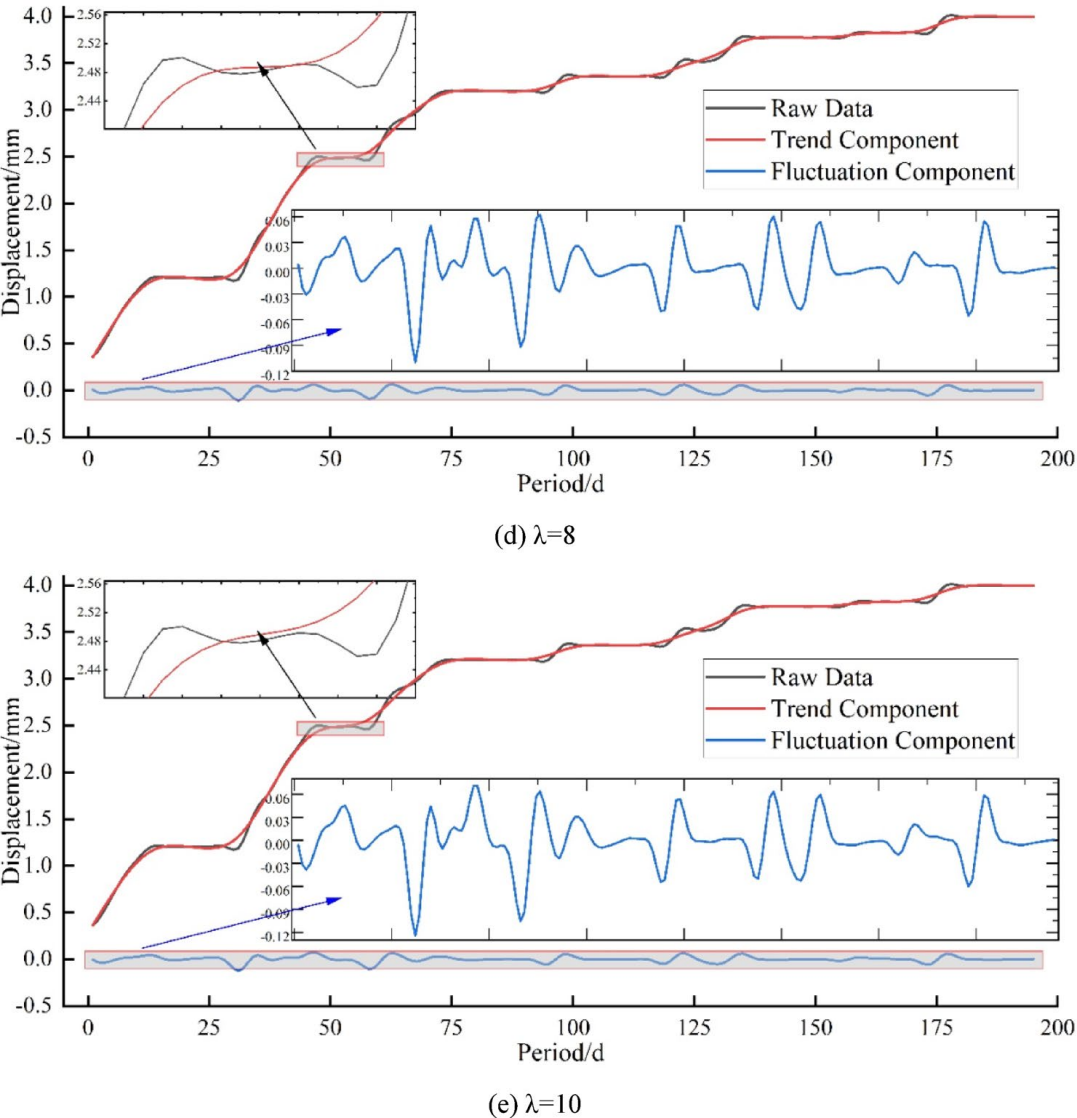
Comparison of Settlement Data Decomposition under. (**a**) λ = 2, (**b**) λ = 4, (**c**) λ = 6, (**d**) λ = 8, (**e**) λ = 10 Different Regularization Parameters.

As shown in Fig. 8, the data decomposition results under five different regularization parameters are compared. When λ = 2, the trend curve and the original data curve almost overlap, and the trend component still contains fluctuation components, indicating incomplete separation of fluctuations. When λ=(4,6,8), the fluctuation components are gradually decomposed. When λ = 10, the trend component perfectly separates the fluctuation component. Huang^48^ pointed out that λ = 10 yields the most reasonable data decomposition. In Fig. 8(e), it can be clearly observed that the influence of fluctuations is significantly eliminated. That is, the trend component obtained through SPA decomposition only shows a noticeable smoothing effect during large fluctuations, and with the increase of the regularization parameter λ, the fluctuation points appear smoother. Therefore, λ = 10 is chosen for the data used in the subsequent experiments.

In summary, VMD decomposition effectively separates the step-like fluctuations (caused by intermittent filling operations and monitoring intervals) into the fluctuation component, resulting in a reconstructed trend component that presents a reasonably smooth and monotonically increasing curve, accurately reflecting the continuous creep characteristics of the soil. In contrast, the trend component obtained from SPA decomposition still contains obvious step-like fluctuations, essentially incorporating construction disturbances into the trend analysis, thereby failing to achieve true modal separation. This fundamental difference indicates that VMD has a significant advantage in analyzing the coupled mechanism of “construction disturbance and soil creep.” The effective separation of each IMF component (trend and fluctuation) makes VMD a superior choice for slope deformation analysis.

### Simulation prediction

#### Determination of the optimal decomposition method

Since the data are time series data, they cannot be shuffled for prediction. Time series prediction is an effective method for settlement data prediction. In this study, the displacement data of the previous 6 days are used to predict the data of the next day, and so on, forming a time series model. The first 80% of the dataset is used as the training set. The optimal hyperparameters learned from the training set are applied to the test set to verify the rationality of the hyperparameter selection for this time series data. The optimal prediction model is determined by comparing the final performance indicators.

The LSSVM model is initialized with a kernel parameter (gamma) of 10 and a penalty parameter (sig2) of 10, with a time delay step set to 6, and employs a single-step prediction approach. The LSTM model is configured with a maximum of 1000 training epochs, an initial learning rate of 0.005, and utilizes the Adam optimization algorithm. Both LSSVM and LSTM models are applied separately to predict the trend and fluctuation components, respectively, to assess the predictive superiority of each model on the different components. To ensure the reliability of the predictions and to avoid randomness in model output, the final prediction results are obtained by averaging five consecutive single-step predictions.

**Fig. 9:**
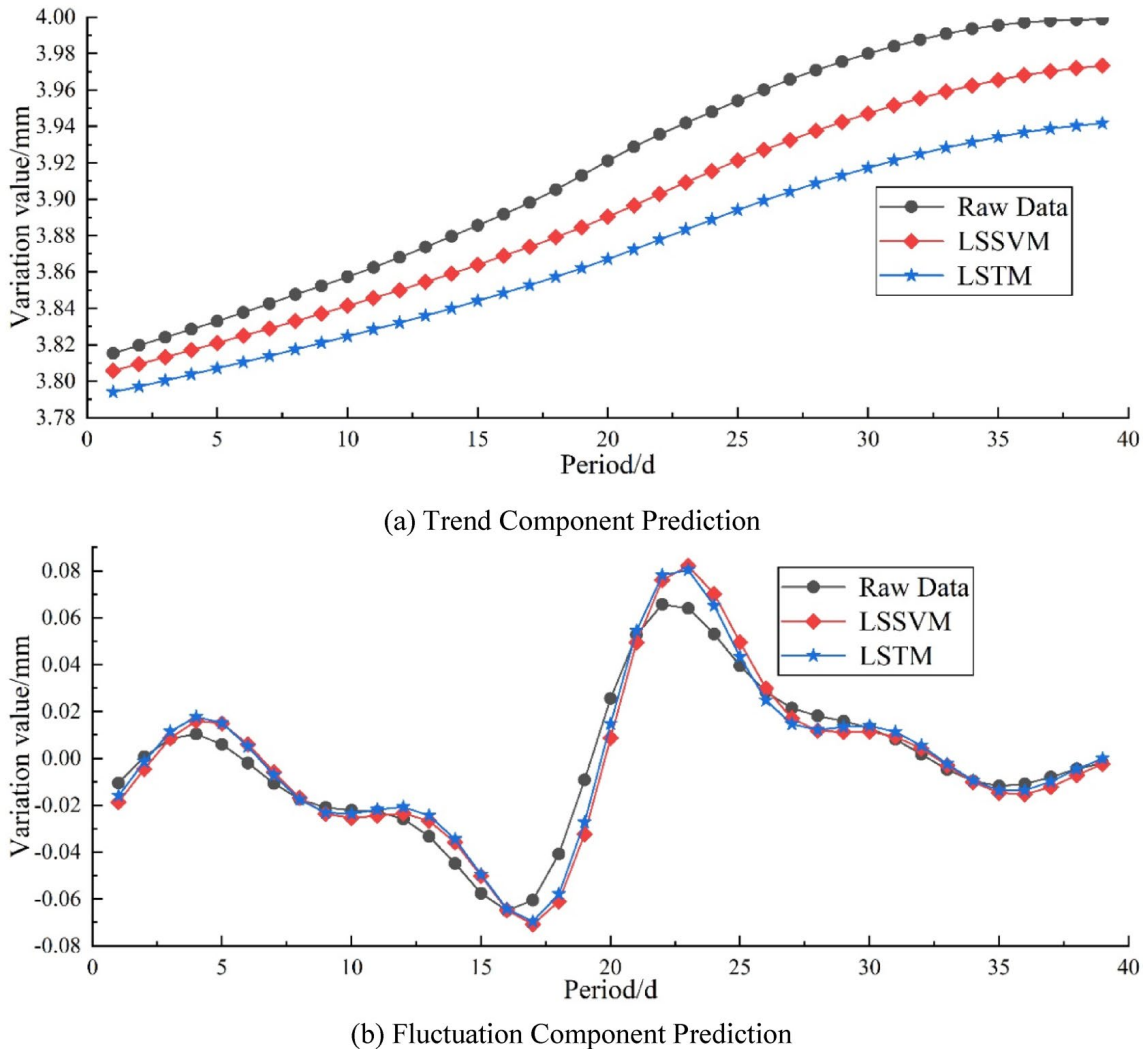
Comparison of Prediction Results for VMD Components. (**a**) Trend Component Prediction, (**b**) Fluctuation Component Prediction.

**Fig. 10:**
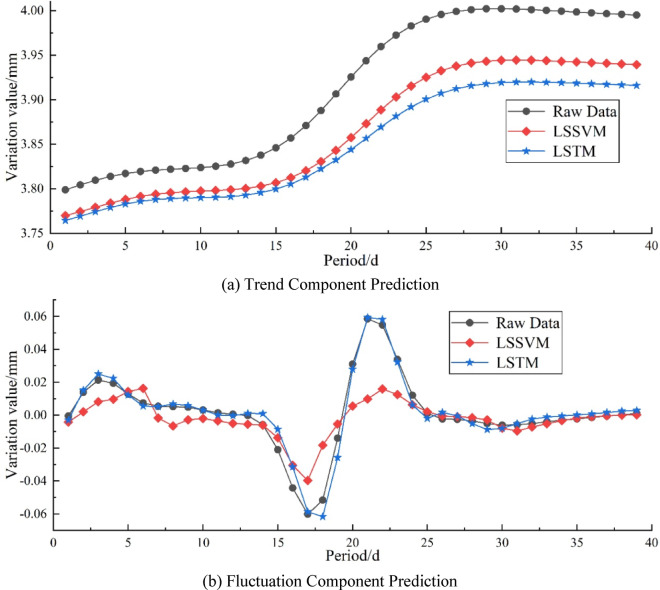
Comparison of SPA component prediction results. (**a**) Trend Component Prediction, (**b**) Fluctuation Component Prediction.

Figures 9 and 10, and Tables 1 and 2 respectively show the prediction curves and performance indicator comparisons of trend and fluctuation components obtained through VMD and SPA decomposition. From the figures, it can be seen that due to differences in decomposition, the results also differ: ①Both methods show good prediction performance on the trend component, and LSSVM fits the original data better than LSTM; ②Regarding the prediction of the fluctuation component, it can be seen that LSSVM is unable to learn the data trend effectively for the fluctuation component decomposed by SPA, but in VMD, it performs similarly to LSTM.

**Fig. 11:**
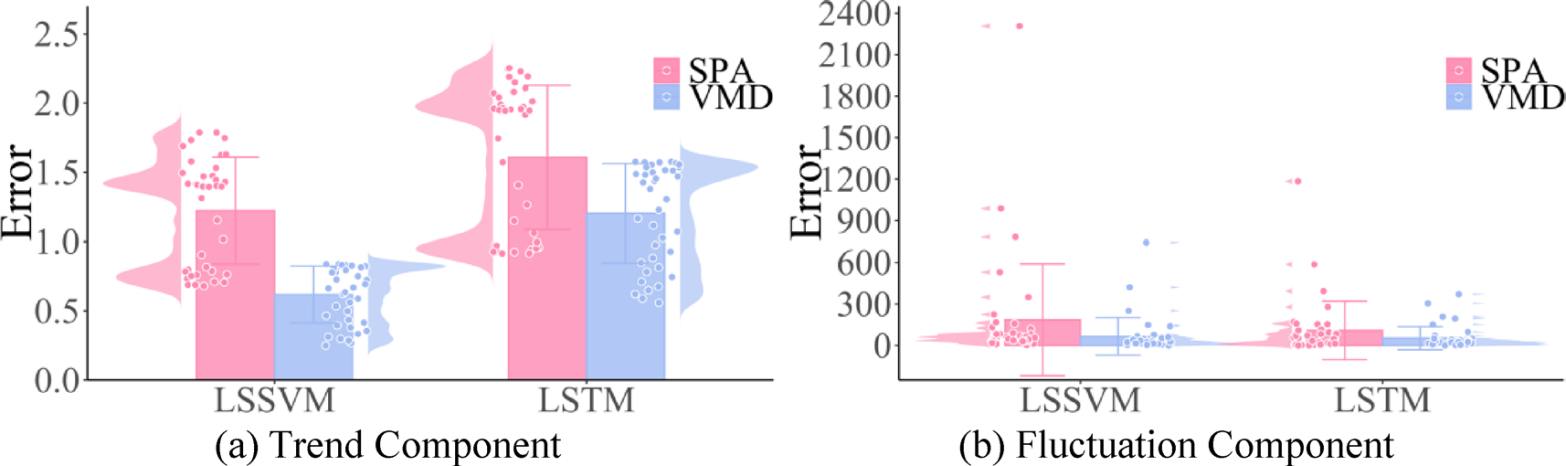
Comparison of Prediction Errors between SPA and VMD.

Figure 11 is a comparison of MAPE root mean square error between VMD and SPA decomposition (unit: %). It shows: ①Both LSSVM and LSTM exhibited excellent prediction performance under VMD decomposition. Compared with the prediction results obtained from SPA decomposition, the prediction errors for the trend component were reduced by 49.18% and 25.00%, while the prediction errors for the fluctuation component were reduced by 62.50% and 56.79%, respectively. ②The error bars represent the uncertainty of the data. The error of the trend component is relatively stable, while the fluctuation component has greater dispersion. From the scatter plot, the fluctuation component has concentrated errors, mostly within 0–250%, but there are still some large deviations from the original data, with the maximum reaching 2300%, indicating the impact of noise in this part of the fluctuation component. In summary, according to the prediction results, the performance of VMD decomposition is better.

**Table 1:** Error comparison of VMD component Predictions.

Metrics	Trend component		Fluctuation component	
	LSSVM	LSTM	LSSVM	LSTM
MAPE/%	0.62	1.20	66.02	52.98
MAE/mm	0.0243	0.0474	0.0062	0.0055
RMSE/mm	0.0257	0.0495	0.0085	0.0073
R^2^/%	82.79	36.07	92.49	94.48
VAF/%	98.24	94.51	92.50	94.55

**Table 2:** Comparison of SPA component prediction errors.

Metrics	Trend component		Fluctuation component	
	LSSVM	LSTM	LSSVM	LSSVM
MAPE/%	1.22	1.60	176.07	122.62
MAE/mm	0.0481	0.0629	0.0089	0.0032
RMSE/mm	0.0506	0.0664	0.0143	0.0045
R2/%	60.84	32.36	59.25	95.78
VAF/%	96.26	92.97	63.42	95.86

#### Comparison of prediction performance for combined components

After decomposing the data into trend and fluctuation components using VMD, in order to determine the most suitable combination for predicting the monitoring data, LSSVM and LSTM were respectively applied to predict the VMD-derived trend and fluctuation components. The predicted components were then recombined, and the predictive performance of the four resulting methods was compared.

**Fig. 12:**
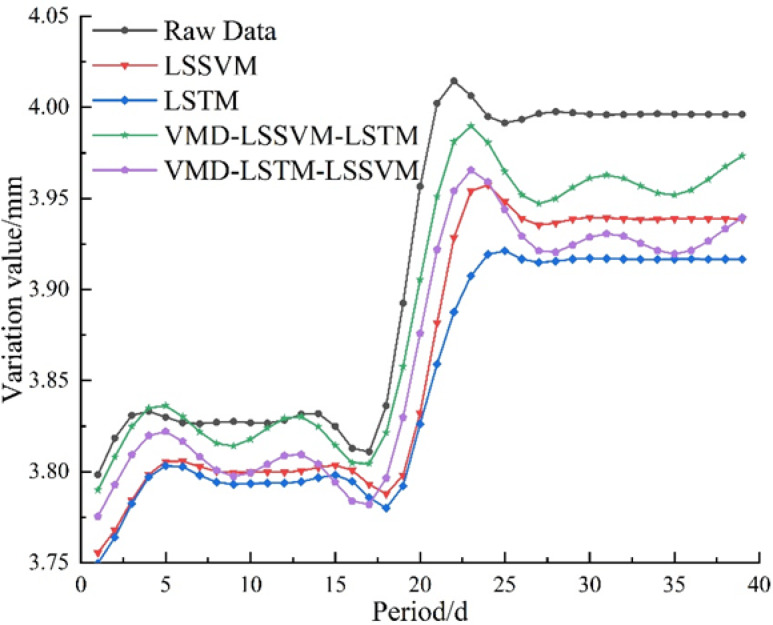
Comparison of Prediction Results for Different Component Combinations.

Figure 12 presents the prediction result curves obtained from the four combined models. Based on these predictions, the evaluation metrics—MAPE, MAE, RMSE, R^2^, and VAF—were calculated. Table 3 compares the prediction accuracy of the four models’ component combinations, with the bolded values representing the VMD-LSSVM-LSTM model, which achieved the lowest values among the groups for the negative indicators, specifically 0.58, 0.0229, and 0.0284, and the highest values for the positive indicators, 88.80 and 95.71. Compared to the other three models, the three negative metrics decreased by 4.09–50.75%, 4.00–50.56%, and 3.64–45.39%, respectively, while the two positive metrics increased by 0.98–42.22% and 0.20–3.63%, respectively. These results clearly demonstrate that the VMD-LSSVM-LSTM model exhibits the best prediction performance.

**Fig. 13:**
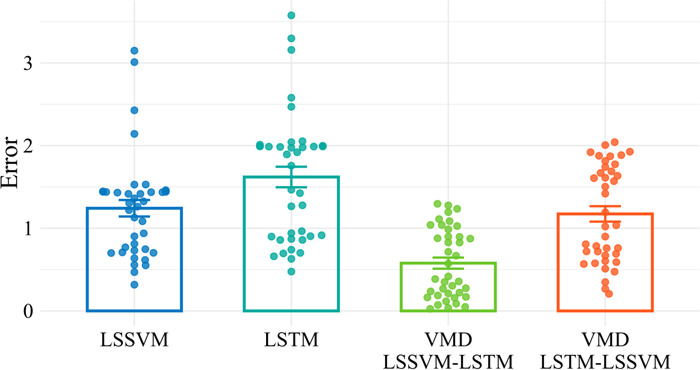
Validation of the Optimal Prediction Combination.

Figure 13 presents a comparison of the mean errors and error ranges among the four models. From the figure, the following observations can be made: (1) The combined prediction models based on decomposition show a significant reduction in maximum error compared to those without decomposition, with prediction errors more concentrated. This indicates that after decomposing the data into trend and fluctuation components, the data become clearer, and errors are less prone to sudden fluctuations, resulting in more stable predictions by the decomposed combined models; (2) Although LSSVM and VMD-LSTM-LSSVM exhibit similar mean error values, the former contains some high-error outliers, suggesting that LSSVM is more susceptible to noise interference; (3) The VMD-LSSVM-LSTM model demonstrates the best error performance, with the smallest and most concentrated mean errors. In summary, the performance ranking of the four methods is as follows: VMD-LSSVM-LSTM > LSSVM > VMD-LSTM-LSSVM > LSTM.

**Table 3:** Comparison of prediction accuracy for component Combination.

Model/Metrics	MAPE/%	MAE/mm	RMSE/mm	*R*^2^/%	VAF/%
VMD-LSSVM	0.60	0.0239	0.0294	87.94	95.52
VMD-LSTM	1.15	0.0453	0.0509	63.92	92.49
VMD-LSSVM-LSTM	**0.58**	**0.0229**	**0.0284**	**88.80**	**95.71**
VMD-LSTM-LSSVM	1.17	0.0464	0.0519	62.44	92.36

### Analysis after optimization of the VMLL model

An appropriate optimization algorithm can significantly improve the predictive performance of the model. In this section, the LSSVM and LSTM models based on the MPA are employed to optimize the kernel function parameter gam and the penalty parameter sig2 of the LSSVM, as well as the maximum number of iterations and the optimal learning rate of the LSTM. The initial values of the hyperparameters are set the same as in Sect. 4.2.1. The population size of the MPA is set to 3, and the maximum number of iterations is set to 100. The search ranges are defined as follows: gam and sig2 of the LSSVM are within [0.01,100], the maximum number of iterations (MaxEpochs) of the LSTM is within [100,1000], and the optimal learning rate (LearnRate) is within [0,0.005].

**Fig. 14:**
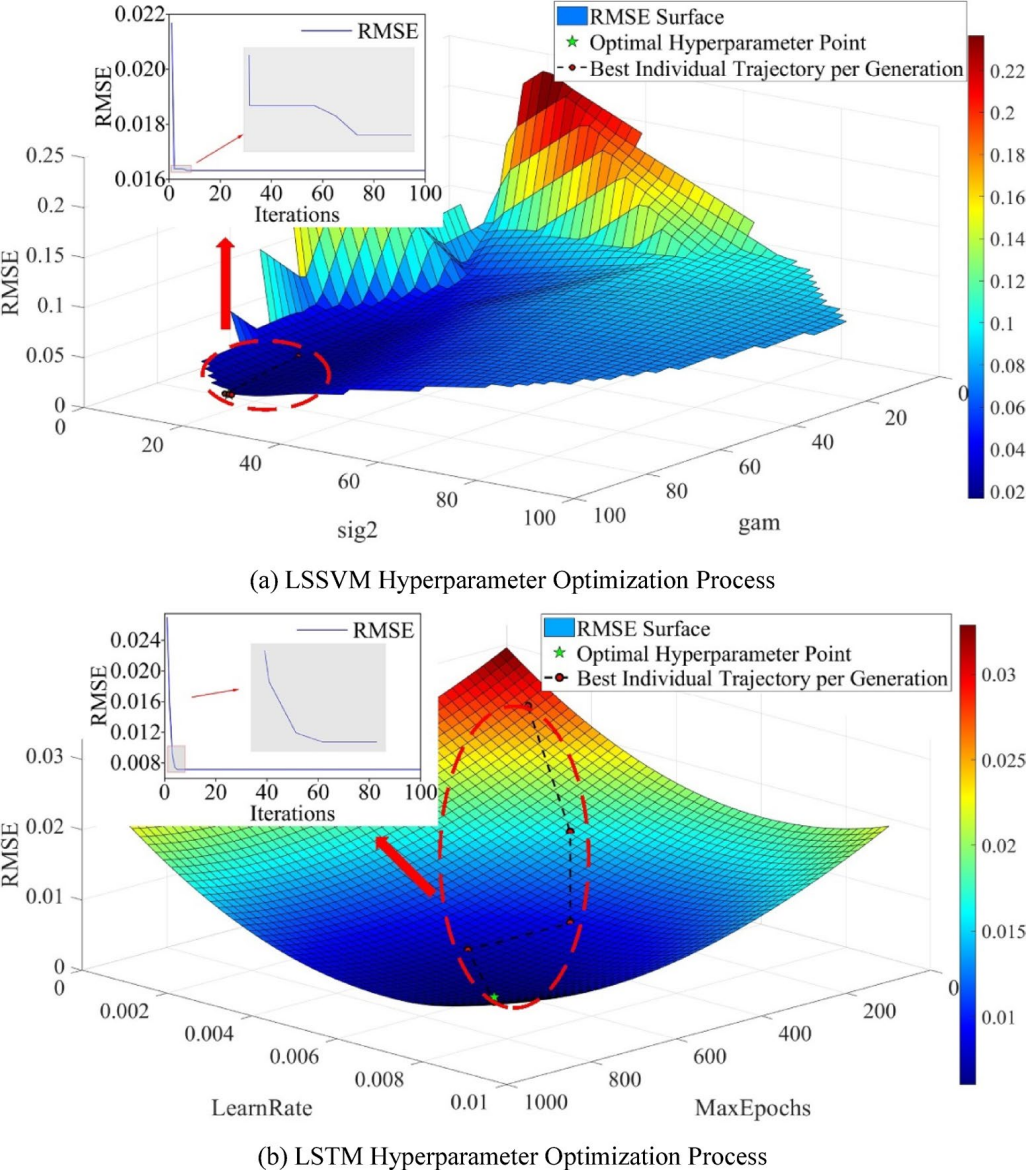
MPA Hyperparameter Optimization. (**a**) LSSVM Hyperparameter Optimization Process, (**b**) LSTM Hyperparameter Optimization Process.

Figure 14 illustrates the hyperparameter optimization process of LSSVM and LSTM using the MPA algorithm, where the optimization trajectory on the RMSE surface plot corresponds to the iteration curve. For the LSSVM model (Fig. 14a), the RMSE surface analysis indicates that the optimal hyperparameter combination (gam = 80.23, sig2 = 20.61) is located at the global minimum (marked with a star), and the iteration curve shows that the algorithm enters a stable convergence phase after the seventh iteration. For the LSTM model (Fig. 14b), the MPA-optimized optimal hyperparameter combination is MaxEpochs = 852 and LearnRate = 0.00823, with the iteration curve showing that the algorithm reaches stable convergence after the fifth iteration. It can be observed that MPA demonstrates fast convergence characteristics in both models, ensuring rapid hyperparameter optimization while avoiding local optima.

**Fig. 15:**
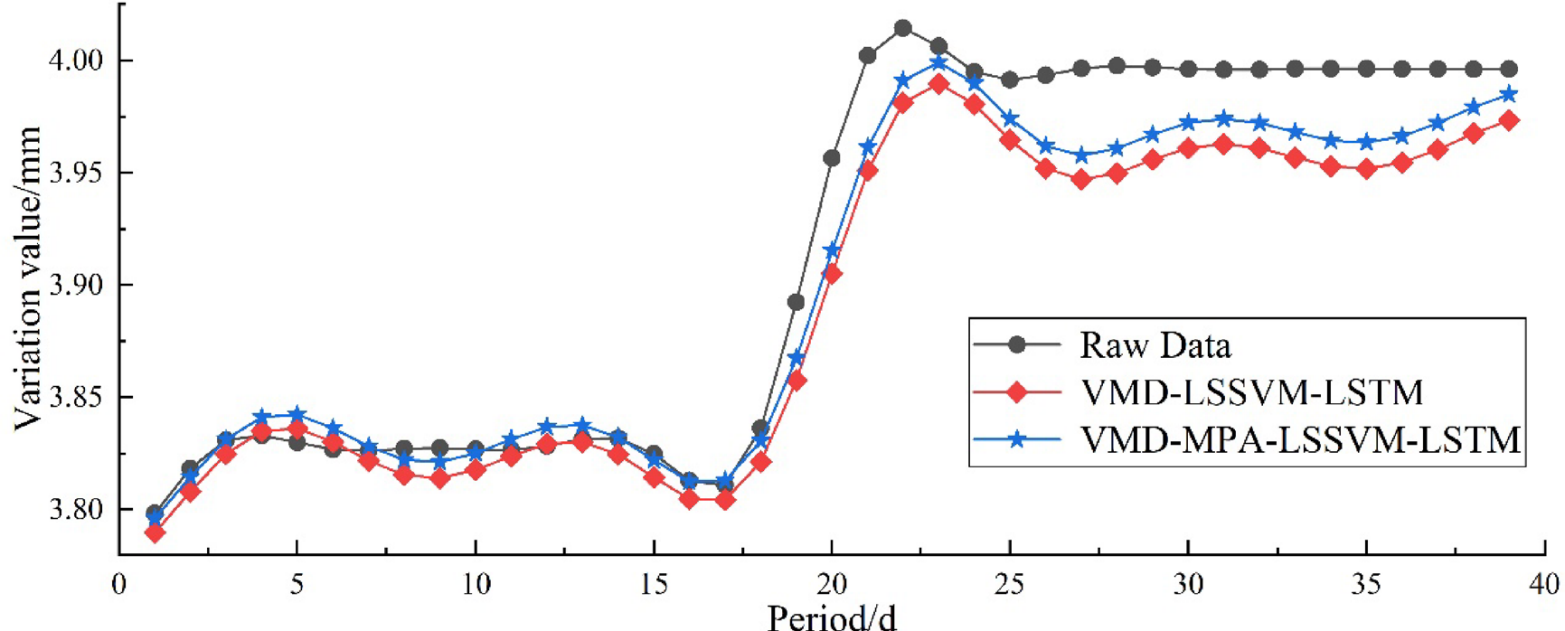
Comparison of Model Predictions after MPA Hyperparameter Optimization.

**Fig. 16:**
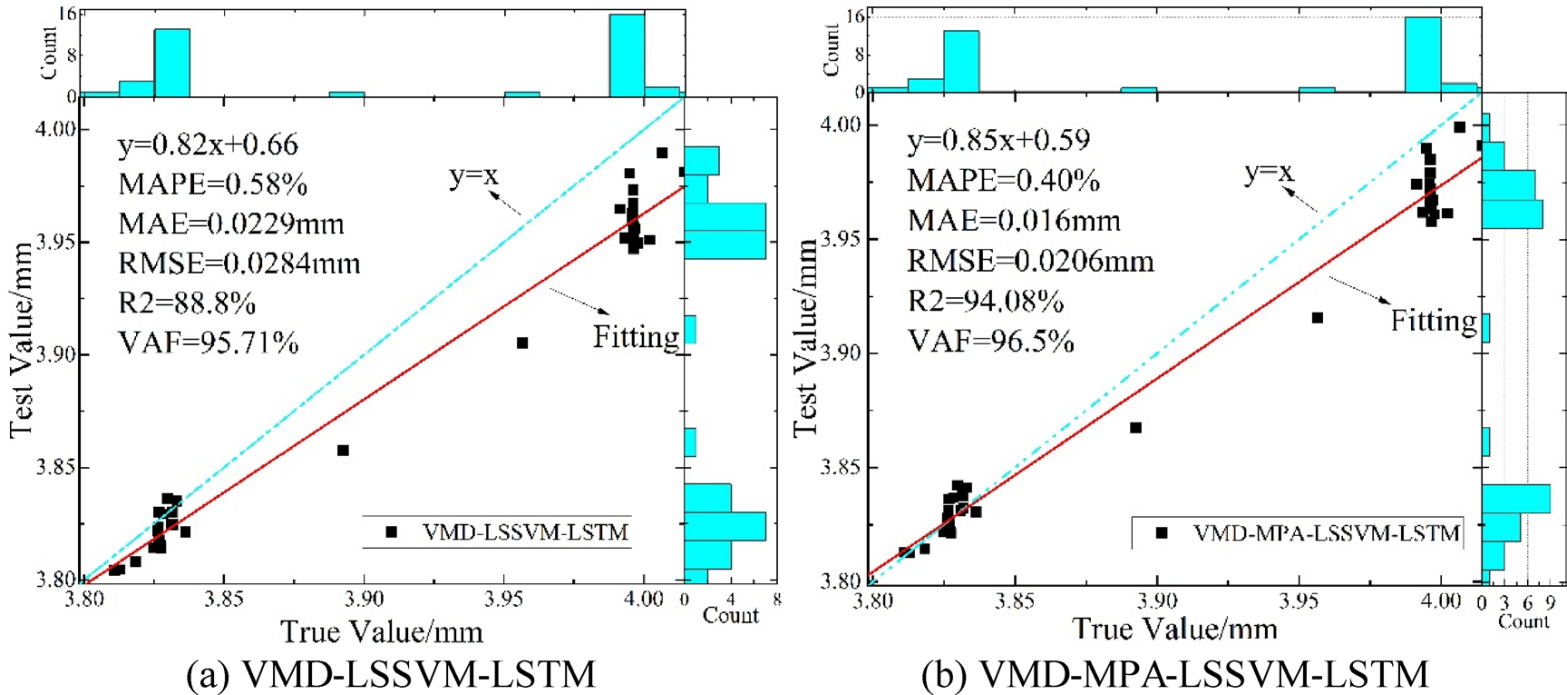
Correlation analysis of two models.

As shown in Fig. 15, after obtaining the optimal hyperparameters through MPA training on the training set, the prediction results on the test set are presented. The optimized hyperparameters are: gam = 80.23, sig2 = 20.61; maximum number of iterations = 52, optimal initial learning rate = 0.00823. It is evident that after hyperparameter optimization, the predicted data fits the original data much more closely, indicating that the MPA marine predators algorithm has good optimization performance. Figure 16 shows the comparison of correlation analysis before and after MPA optimization. The red curve in the figure represents the fitting regression result between the true values and predicted values on the test set. The fitted curves of both methods are close to y = x, indicating good model adaptability to the trend and fluctuation components. Among them, the fitted curve of the VMD-MPA-LSSVM-LSTM model is closer to y = x, suggesting smaller errors and more accurate prediction results. Moreover, the figure shows that the error metrics MAPE, MAE, RMSE, R^2^, and VAF are 0.40%, 0.016 mm, 0.0206 mm, 94.08%, and 96.5%, respectively. The blue histograms on the top and right sides of the figure show the number of points for true and predicted values in each interval, indicating that the VMD-MPA-LSSVM-LSTM model achieves higher point overlap and thus has the best prediction performance.

**Fig. 17:**
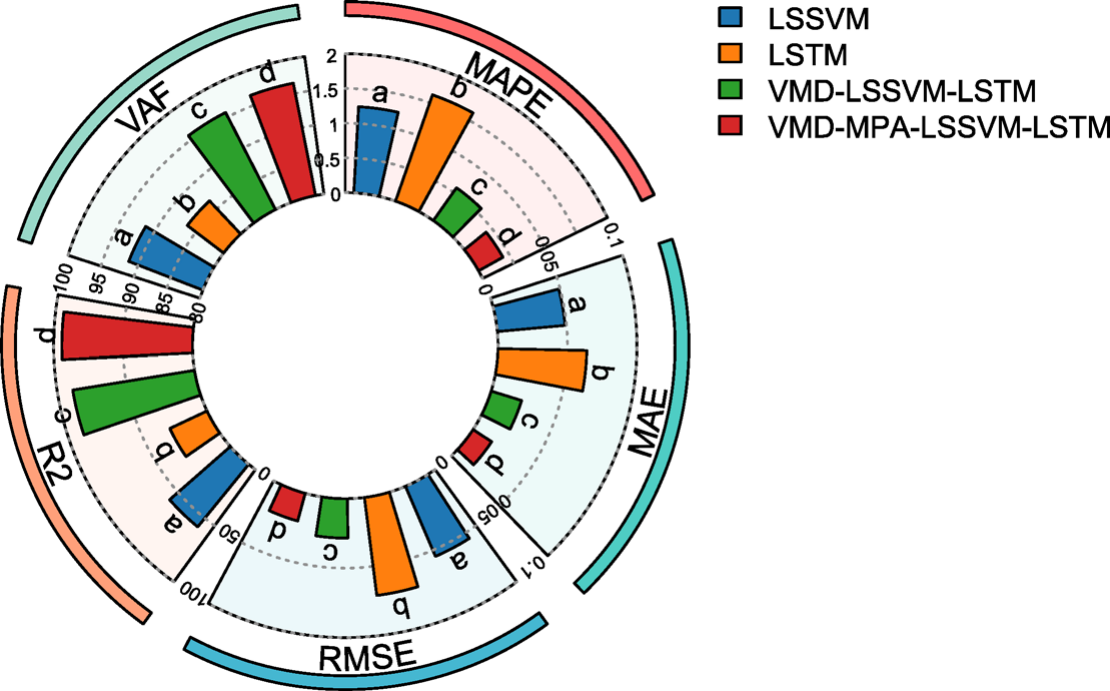
Model Performance Comparison.

To more clearly compare the model performance before and after optimization, Table 4 presents the error performance metrics for each model, accompanied by a polar bar chart shown in Fig. 17, which provides a more intuitive comparison of the predictive results across multiple models. As shown in Table 4; Fig. 17: (1) The three negative indicators—MAPE, MAE, and RMSE—demonstrate substantial reductions under the VMD-MPA-LSSVM-LSTM model compared to the other three models, with MAPE decreasing by 30.41–67.62%, MAE decreasing by 30.38–67.40%, and RMSE decreasing by 27.32–71.00%; (2) The two positive indicators—R² and VAF—show significant improvements, with R² increasing by 5.95–217.51%(R^2^_VMD − MPA−LSSVM−LSTM_=94.08%, R^2^_LSTM_ = 94.08%) and VAF increasing by 0.83–11.48%. Clearly, among the four models, VMD-MPA-LSSVM-LSTM achieves the best performance on both positive and negative metrics, demonstrating the rationality and advantages of the VMD decomposition and MPA optimization algorithm.

**Table 4:** Prediction performance metrics for Disp.P1.

Model/Metrics	MAPE/%	MAE/mm	RMSE/mm	*R*^2^/%	VAF/%
LSSVM	1.2423	0.0489	0.0549	58.10	91.45
LSTM	1.6204	0.0639	0.0711	29.63	86.56
VMD-LSSVM-LSTM	0.5780	0.0229	0.0284	88.80	95.71
VMD-MPA-LSSVM-LSTM	0.4022	0.0160	0.0206	94.08	96.50

### Analysis of robustness with small sample sizes

In time series prediction tasks, the size of the training data often directly affects the model’s predictive performance. Small-sample data refers to the limited amount of valid displacement monitoring data obtained due to engineering constraints. To explore the effective modeling conditions under limited data and verify the predictive stability of the VMLL model with different small-sample data volumes, this section gradually reduces the training sample size. A staged time series segmentation method (50, 100, 150 samples) is used with a fixed test set (the last 39 data points). taking the previous prediction results based on 156 samples as a reference, the performance of the VMLL model under different data scales is evaluated, and its engineering applicability is analyzed.

**Fig. 18:**
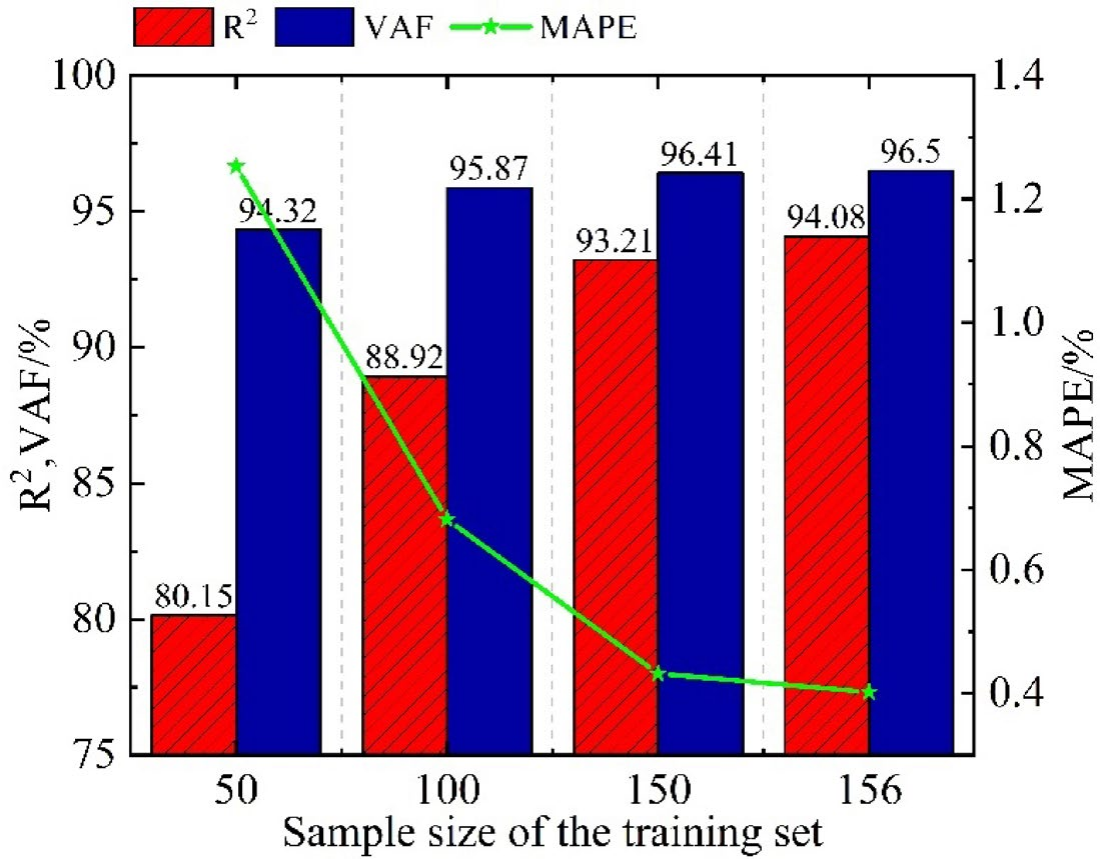
Comparison of metrics under different training sample sizes.

Table 5 presents the detailed changes in the performance metrics of the VMLL model under different training sample sizes, and Fig. 18 provides a comparison of the metrics across these varying training sample volumes. The experimental data indicate that as the training sample size increases, the model’s performance exhibits a steadily improving trend. Specifically, the three error metrics—MAPE, MAE, and RMSE—continuously decrease as the training sample size increases, while the two positive indicators, R² and VAF, steadily improve with the increasing sample size; moreover, even under the minimum sample size condition (50 samples), the model can still maintain a high performance level with R²>80% and VAF > 94%, demonstrating strong fundamental predictive capability; furthermore, the results indicate that when the sample size reaches 156, all model performance metrics achieve their optimal values.

**Table 5:** Prediction evaluation metrics for different training sample sizes.

Training sample size	MAPE/%	MAE/mm	RMSE/mm	*R*^2^/%	VAF/%
156	0.402	0.016	0.021	94.08	96.50
150	0.432	0.017	0.022	93.21	96.41
100	0.682	0.025	0.028	88.92	95.87
50	1.253	0.036	0.044	80.15	94.32

The experimental results indicate that the VMLL model demonstrates remarkable adaptability to small sample sizes. Specifically, first, under extreme data scarcity (50 samples), the model still maintains relatively high prediction accuracy; second, as the sample size increases, the model’s performance stabilizes, and under the full training set, the model reaches a performance saturation state, with all evaluation metrics converging to their optimal levels.

#### Model validation

To avoid the model overfitting to a single data series, vertical settlement data from monitoring slope 1, as well as horizontal displacement and vertical settlement data from monitoring slope 2, were selected as the validation set. The VMD-MPA-LSSVM-LSTM model was used for prediction and compared against LSSVM, LSTM, and VMD-LSSVM-LSTM models for comprehensive evaluation. The MAPE, MAE, RMSE, R², and VAF indicators of each model were calculated to verify the prediction performance of the model on the new dataset and to assess its generalization and robustness. The structure and parameters of each model were kept the same as those used for predicting horizontal displacement on slope 1.

##### Data decomposition

**Fig. 19:**
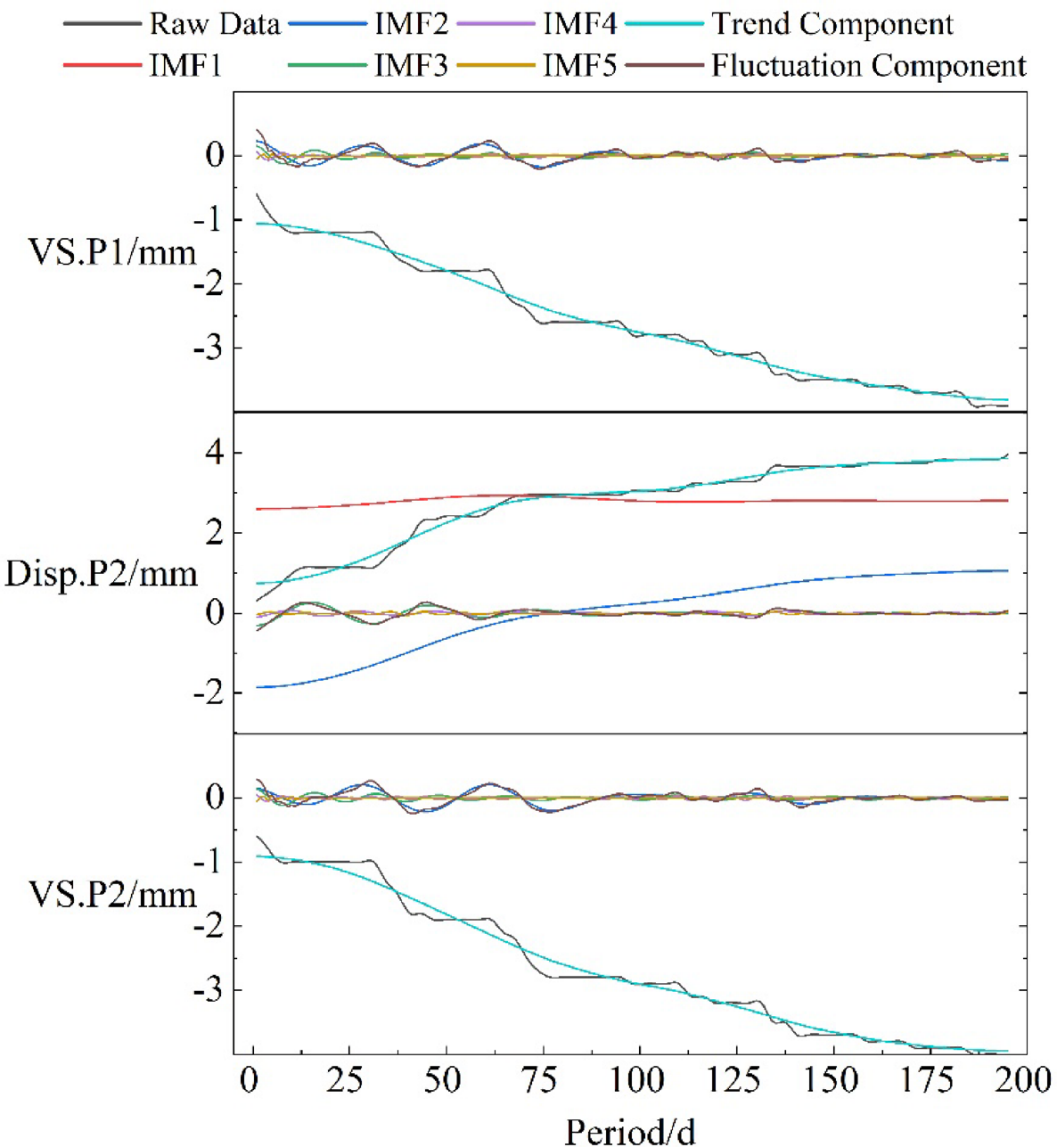
Data decomposition.

By utilizing VMD to decompose VS.P1, Disp.P2, and VS.P2, five components are obtained; through effective combination of these components, the trend term and fluctuation term are derived. As shown in Fig. 19, both VS.P1 and VS.P2 use IMF1 as the trend term, with the sum of the other four components as the fluctuation term; Disp.P2 uses the sum of IMF1 and IMF2 as the trend term, with the sum of the other three components as the fluctuation term.

##### Prediction analysis

**Fig. 20:**
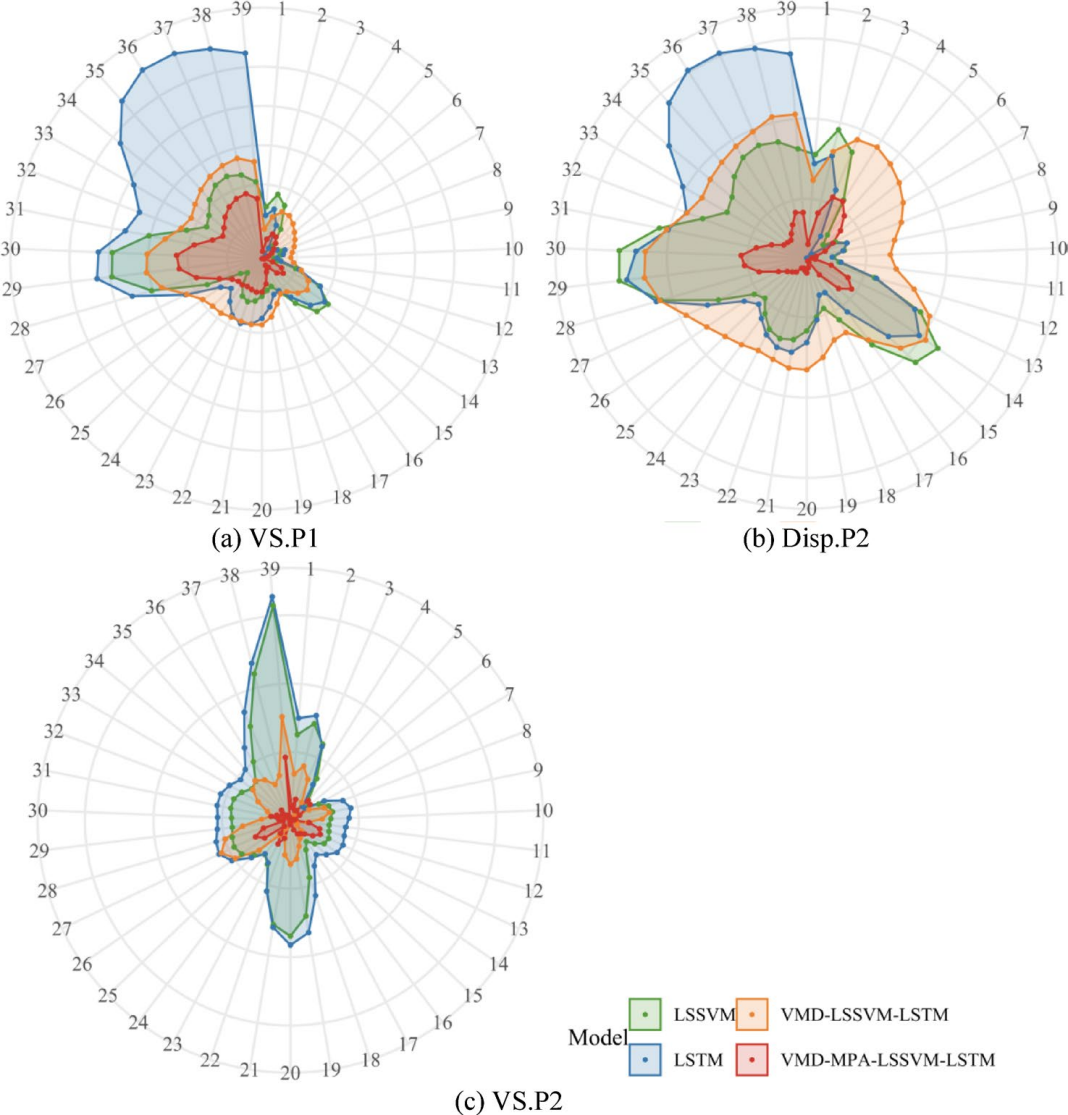
Comparison of MAPE errors.

As shown in Fig. 20, the error area charts of predictions using LSSVM, LSTM, VMD-LSSVM-LSTM, and VMD-MPA-LSSVM-LSTM models reveal the following from three prediction perspectives: ①LSTM exhibits the most significant error fluctuations, indicating that among these four models, LSTM’s prediction errors vary the most; ②VMD-MPA-LSSVM-LSTM shows the smallest error fluctuation range. Overall, VMD-MPA-LSSVM-LSTM has the smallest error area, meaning it has the narrowest overall error fluctuation and better performance. At individual prediction points, due to different data learning characteristics, some points inevitably have larger errors than other models, but all remain within a controllable error range.

To verify the rationality of the error results, SPSS was used to perform paired t-tests comparing the prediction errors of the VMD-MPA-LSSVM-LSTM model as the reference against the other three models. Table 6 shows the results of the paired t-tests, revealing that the mean differences and t-values for all three perspectives are less than 0, and the p-values are all below 0.05. This confirms the authenticity and reliability of the prediction errors and demonstrates that overall, the VMD-MPA-LSSVM-LSTM model has smaller errors and better performance than the other three models.

**Table 6:** Results of paired t-Test.

	Comparison model	Mean difference	95% confidence interval	t	*p*
VS.P1	LSSVM	-5.78	[-0.74, -0.41]	-7.101	0.000
	LSTM	-1.29	[-1.70, -0.88]	-6.294	0.000
	VMD-LSSVM-LSTM	-8.00	[-0.84, -0.77]	-45.982	0.000
Disp.P2	LSSVM	-0.70	[-0.87, -0.52]	-8.055	0.000
	LSTM	-0.86	[-1.03, -0.68]	-9.619	0.000
	VMD-LSSVM-LSTM	-0.27	[-0.37, -0.16]	-4.965	0.000
VS.P2	LSSVM	-0.77	[-0.91, -0.62]	-10.678	0.000
	LSTM	-0.88	[-1.13, -0.64]	-7.392	0.000
	VMD-LSSVM-LSTM	-1.10	[-1.15, -0.06]	-45.795	0.000

To more clearly compare the advantages and disadvantages of the different models, Table 7 presents five evaluation metrics along with the average values for the three datasets. The bolded numbers in the table represent the best prediction performance for each metric. As shown in the table, the VMD-MPA-LSSVM-LSTM model performs the best across all performance indicators. Taking the average values of the three datasets as reference, the MAPE, MAE, RMSE, R², and VAF are 0.6207, 0.0238, 0.0273, 90.33, and 96.81, respectively. Compared to the other three models, these results represent improvements of 37.23–78.12%, 37.03–78.18%, 36.07–75.48%, 28.18–438.71%, and 0.61–42.43%, respectively. This demonstrates that the VMD-based data decomposition combined with MPA-optimized LSSVM and LSTM models can more effectively perform composite prediction of slope deformation data. Based on a comprehensive comparison of the evaluation metrics, whether from an individual perspective or in terms of average values, the VMD-MPA-LSSVM-LSTM model is the most accurate prediction model.

**Table 7:** Prediction result Metrics.

	Model / Metric	MAPE/%	MAE/mm	RMSE/mm	*R*^2^/%	VAF/%
VS.P1	LSSVM	1.5525	0.0586	0.0682	67.75	91.52
	LSTM	2.2638	0.0862	0.1101	16.12	67.59
	VMD-LSSVM-LSTM	1.7749	0.0668	0.0719	64.24	95.13
	VMD-MPA-LSSVM-LSTM	**0.9745**	**0.0369**	**0.0436**	**86.84**	**96.27**
Disp.P2	LSSVM	0.9361	0.0357	0.0414	41.22	84.83
	LSTM	1.0946	0.0417	0.0469	24.69	84.21
	VMD-LSSVM-LSTM	0.5042	0.0192	0.0227	82.29	94.93
	VMD-MPA-LSSVM-LSTM	**0.2395**	**0.0091**	**0.0115**	**95.45**	**95.51**
VS.P2	LSSVM	1.4129	0.0553	0.0594	45.09	92.70
	LSTM	1.5335	0.0602	0.0680	27.87	84.40
	VMD-LSSVM-LSTM	1.7474	0.0682	0.0691	25.58	98.03
	VMD-MPA-LSSVM-LSTM	**0.6481**	**0.0253**	**0.0269**	**88.71**	**98.66**
Average	LSSVM	1.3005	0.0499	0.0563	51.35	89.68
	LSTM	1.6306	0.0627	0.0750	22.89	78.73
	VMD-LSSVM-LSTM	1.3422	0.0514	0.0546	57.37	96.03
	VMD-MPA-LSSVM-LSTM	**0.6207**	**0.0238**	**0.0273**	**90.33**	**96.81**

Significant values are in bold.

## Conclusion

### Research analysis and discussion

This study establishes a combined slope deformation prediction model based on data decomposition and optimization algorithms, namely the VMLL model. By predicting the horizontal displacement and vertical settlement data of the Hongtuya high-fill embankment, and further comparing and validating the effectiveness of different model predictions, the optimal prediction model was obtained. The research results indicate that:

1. The VMD method effectively separated the trend and fluctuation components of the displacement data. Using LSSVM and LSTM for prediction, and comparing the results with those obtained from SPA decomposition, the prediction errors of the trend component were reduced by 49.18% and 25.00%, respectively, while those of the fluctuation component were reduced by 62.50% and 56.79%, respectively, indicating that VMD achieved superior decomposition performance.
2. The combined prediction approach using LSSVM and LSTM demonstrated outstanding performance. Compared with other methods, the VMD-LSSVM-LSTM model improved all evaluation metrics by 4.09–50.75%, 4.00–50.56%, 3.64–45.39%, 0.98–42.22%, and 0.20–3.63%, respectively. The results indicate that LSSVM performs better in predicting the trend component, whereas LSTM shows greater advantages in predicting the fluctuation component.
3. With the aid of MPA optimization, the prediction accuracy of the VMLL model was significantly improved, achieving MAPE, MAE, RMSE, R², and VAF values of 0.4022%, 0.0160 mm, 0.0206 mm, 94.08%, and 96.50%, respectively. Compared with the other three models, these represent improvements of 30.41–67.62%, 27.32–71.00%, 5.95–217.51%, and 0.83–11.48%, respectively. These results provide strong evidence of the effectiveness of the MPA optimization strategy.
4. Finally, prediction validation on three independent monitoring datasets (VS.P1, Disp.P2, and VS.P2) demonstrated that the VMLL model maintained stable prediction performance, with MAPE, MAE, RMSE, R², and VAF values of 0.6207%, 0.0238 mm, 0.0273 mm, 90.33%, and 96.81%, respectively.

### Research contributions and prospects

The VMLL model demonstrates significant application potential in the Hongtuyao slope. By dynamically predicting real-time monitoring data, the model can capture subtle trends in slope displacement. Combined with engineering early-warning thresholds, it enables the timely identification of potential landslide risks and provides reliable warning signals for engineering personnel.

The main theoretical contributions of this study can be summarized in three aspects: ①VMD effectively decomposes the data, with LSSVM being well-suited for trend prediction and LSTM excelling in fluctuation prediction; ②The use of the MPA algorithm enables multi-objective optimization, achieving collaborative tuning of the two models; ③A novel “decomposition–component-wise prediction–integration” framework is proposed, offering a new technical approach for slope deformation prediction.

Outlook: ①Integrate multi-source monitoring data, such as meteorological and groundwater level data, to improve the physical rationality and prediction accuracy of the model; ②Conduct cross-regional and cross-geological engineering validation, while optimizing algorithm efficiency to achieve lightweight and real-time monitoring capabilities; ③Combine digital twin technology to realize intelligent management of slope engineering throughout its entire lifecycle.

## Data Availability

The datasets used and/or analysed during the current study are available from the corresponding author on reasonable request.
